# Nuclear shape, protrusive behaviour and *in vivo* retention of human bone marrow mesenchymal stromal cells is controlled by Lamin-A/C expression

**DOI:** 10.1038/s41598-019-50955-x

**Published:** 2019-10-07

**Authors:** Yvonne L. Dorland, Anne S. Cornelissen, Carlijn Kuijk, Simon Tol, Mark Hoogenboezem, Jaap D. van Buul, Martijn A. Nolte, Carlijn Voermans, Stephan Huveneers

**Affiliations:** 1 Sanquin Research and Landsteiner Laboratory, Department of Molecular and Cellular Hemostasis, Amsterdam UMC, University of Amsterdam, Amsterdam, The Netherlands; 2 Sanquin Research and Landsteiner Laboratory, Department of Hematopoiesis, Amsterdam UMC, University of Amsterdam, Amsterdam, The Netherlands; 3 Amsterdam UMC, University of Amsterdam, Department of Medical Biochemistry, Amsterdam Cardiovascular Sciences, Amsterdam, The Netherlands

**Keywords:** Cell biology, Nuclear organization, Mesenchymal stem cells

## Abstract

Culture expanded mesenchymal stromal cells (MSCs) are being extensively studied for therapeutic applications, including treatment of graft-versus-host disease, osteogenesis imperfecta and for enhancing engraftment of hematopoietic stem cells after transplantation. Thus far, clinical trials have shown that the therapeutic efficiency of MSCs is variable, which may in part be due to inefficient cell migration. Here we demonstrate that human MSCs display remarkable low migratory behaviour compared to other mesodermal-derived primary human cell types. We reveal that specifically in MSCs the nucleus is irregularly shaped and nuclear lamina are prone to wrinkling. In addition, we show that expression of Lamin A/C is relatively high in MSCs. We further demonstrate that *in vitro* MSC migration through confined pores is limited by their nuclei, a property that might correlate to the therapeutic inefficiency of administered MSC *in vivo*. Silencing expression of Lamin A/C in MSCs improves nuclear envelope morphology, promotes the protrusive activity of MSCs through confined pores and enhances their retention in the lung after intravenous administration *in vivo*. Our findings suggest that the intrinsic nuclear lamina properties of MSCs underlie their limited capacity to migrate, and that strategies that target the nuclear lamina might alter MSC-based cellular therapies.

## Introduction

Bone marrow-derived mesenchymal stromal cells (MSCs^1^), are promising candidates for cellular therapies because of their regenerative and immunomodulatory potential^2–5^. Clinical efficacy of administered MSCs has been demonstrated in a variety of diseases including graft-versus-host disease (GvHD)^6^, non-union fractures^7^ and osteogenesis imperfecta^8^. However, the percentage of administered MSCs that migrates towards and engrafts affected tissues (e.g. bone, intestine or inflamed tissue) in both animal models and patients remains very low^5,9–11^. Migratory behaviour can vary between primary cells, depending on intrinsic cellular properties and local signals from the microenvironment. Two features that also determine the dynamics and efficiency of administered cells in cellular therapies^12–16^. The limited migration of culture expanded MSCs might be partly explained by the low intrinsic expression of homing receptors or the loss of chemokine receptors upon culturing^17^. Moreover, MSCs are natively strong adherent cells that rely on signals from the extracellular matrix for migration, polarization and survival. MSCs that are administered to the bloodstream have to survive being in suspension and lack the signals from the tissue microenvironment to direct migration. To explore approaches to improve MSC-based cellular therapies, previous research has focused on increasing the expression of chemokine receptors or decreasing availability of adhesion molecules (reviewed in^18,19^). However, most of these migration-promoting methods have not yet been validated *in vivo* and therefore a correlation between MSC homing and clinical outcome still needs to be demonstrated^10,18^.

Unlike haematopoietic cells, MSCs are not well adapted to circulate through the vasculature. The average lumen size within the human vasculature ranges from 30 mm in the vena cava to 8 µm in the smallest capillaries^20^, whereas MSCs in suspension have an average diameter of 15–30 µm^21,22^. Also, in contrast to hematopoietic cells such as erythrocytes (no nucleus) or granulocytes (lobular/flexible nucleus), MSCs are not specialized to squeeze their proportionally large nuclei through restricted spaces such as small capillaries or to transmigrate through the blood vessel wall to invade tissue^23^. Indeed, *in vivo* tracking studies in animal models demonstrated that the majority of intravenously injected MSCs are cleared from the circulation within 5 minutes. MSC first become entrapped in the small capillaries of the lung vasculature before being detected in the liver, kidney and spleen^22,24,25^. Virtually no MSCs reach the bone marrow after intravenous administration into irradiated mice, whereas intra-bone marrow transplantation of MSCs results in engraftment throughout the entire injected bone^26^.

Migration through tissue and sensing of the microenvironment tightly depends on the rigidity, shape and anchoring of the nucleus within the cytoskeleton^12,27–29^. These properties are controlled by the nuclear lamina proteins Lamin A/C and Lamin B1^30^ and through coupling of the nuclear envelope to the cytoskeleton via the LINC complex^31^. While sensing of the substrate rigidity through nucleus-cytoskeletal coupling has been widely studied in the context of MSC differentiation^32^, the role of nuclear lamina in MSC migration has not been addressed in great detail. Here we compared the migratory behaviour of MSCs with other primary human cell types derived from mesodermal origin. We uncover that the specific slow migration of MSCs is correlated with differing nuclear properties. Moreover, we find that the nucleus of MSCs limits their migration through confined spaces, a characteristic that might explain their low migration and homing capacity *in vivo*. Finally, we show that silencing expression of Lamin A/C promotes the protrusive capacity of MSCs, suggesting that targeting of the MSC nuclear envelope might change therapeutic efficiency of MSCs.

## Results

### MSC migrate slowly

**Figure 1:**
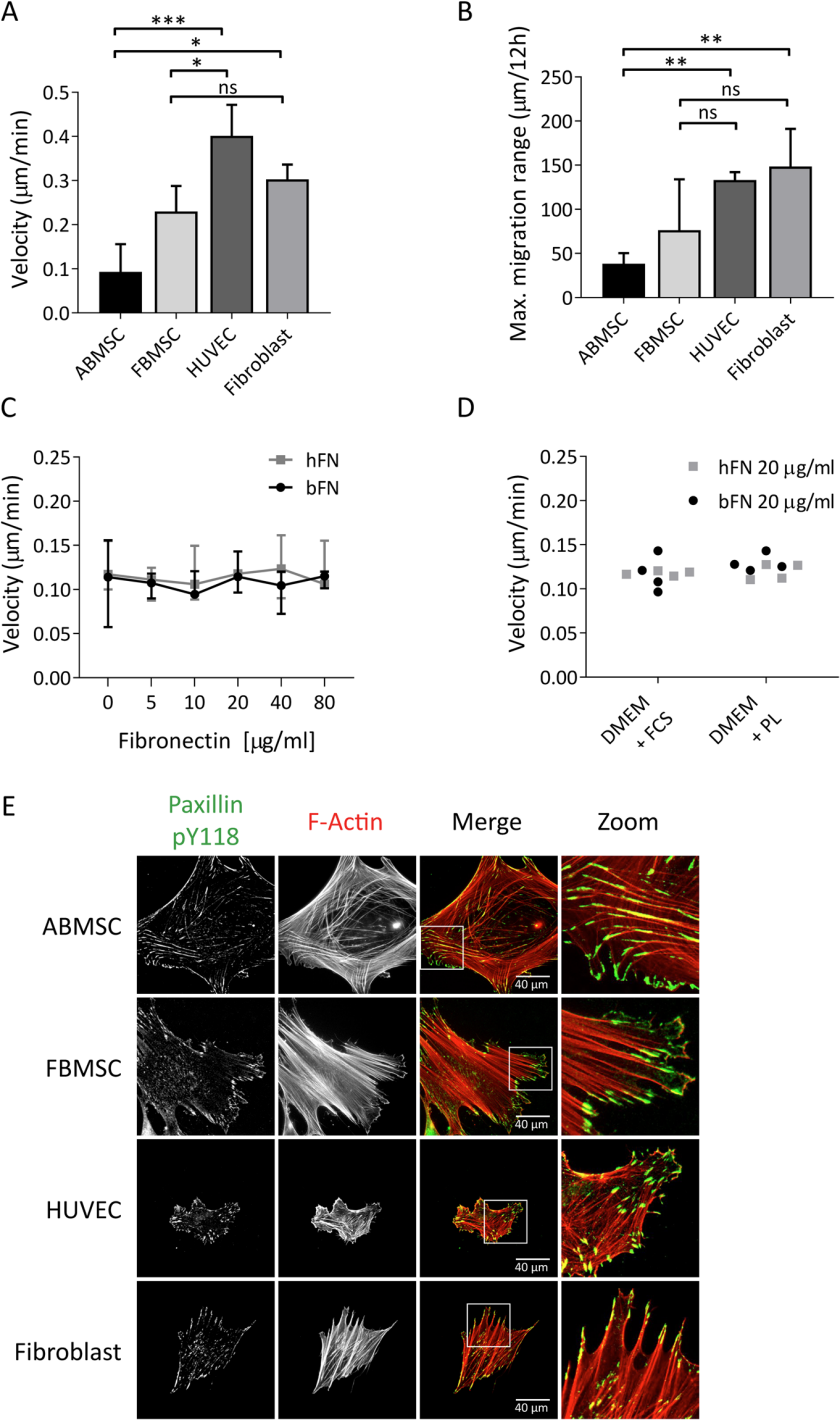
Cell migration and focal adhesion formation in MSCs compared to other primary mesodermal-derived adherent cells. (**A**) Average migration velocity of ABMSC compared to FBMSC, HUVEC and fibroblasts, measured by live cell phase contrast microscopy. Images were obtained every 30 minutes for a total of 12 hours at 37 °C, 5% CO_2_ and migration was tracked using ImageJ. Median ± range. n = 4 independent experiments including 30 cells each. ns = non-significant, *p < 0.05, ***p < 0.001 (Kruskal-Wallis, multiple comparisons uncorrected Dunn’s test). (**B**) Maximum absolute travel distance from point origin of ABMSC, FBMSC, HUVEC and fibroblasts within 12 hours. Median ± range. n = 4 independent experiments, including 30 cells each. ns = non-significant, **p < 0.01 (Kruskal-Wallis, multiple comparisons uncorrected Dunn’s test). (**C**) Graph shows ABMSC migration velocity on varying concentrations of human-derived fibronectin (hFN) and bovine-derived fibronectin (bFN). Images were obtained every 30 minutes for a total of 12 hours at 37 °C, 5% CO_2_ and migration was tracked using ImageJ. Median ± range. n = 4 independent experiments comprising cells from 4 different donors, analysis includes 15 cells per condition. Spearman correlation test. (**D**) Average migration velocity of ABMSCs cultured in either Dulbecco’s Modified Eagle’s Medium (DMEM) with 10% fetal calf serum (FCS) or with 5% Platelet lysate (PL) and cultured on 20 µg/ml hFN or bFN. n = 4 independent experiments comprising cells from 4 different donors, analysis includes 15 cells per condition. (Kruskal-Wallis, multiple comparisons uncorrected Dunn’s test). (**E**) Representative widefield immunofluorescence (IF) images of ABMSC, FBMSC, HUVEC and fibroblasts stained for Y118 phosphorylated Paxillin (green) and F-actin (red). Magnified images from regions of interest (ROI) show focal adhesions. Scalebar: 40 μm.

The migratory behaviour of cultured MSCs used for cellular therapies has been previously studied in response to various stimuli^17,33–35^, and tends to be particular slow^12^. To investigate the migration capacity of human MSCs in direct comparison to other primary mesodermal derived adherent cells, we used adult bone marrow-derived MSC (ABMSC), fetal human bone marrow-derived MSC (FBMSC), human umbilical vein endothelial cells (HUVECs) and human skin fibroblasts. Cells were plated sparsely on 20 μg/ml bovine fibronectin-coated substrates, and subsequently imaged over a time course of 12 hours using phase contrast microscopy. Tracking of single cell migration showed that the average migration velocity of ABMSCs under these conditions was 0.1 µm/min and of FBMSCs was 0.24 µm/min (Fig. 1A and Supplemental Movie 1), which is similar to migration velocities of MSCs reported previously in literature^36,37^. By contrast, the average migration speed of HUVECs and human fibroblasts was higher compared to ABMSC (Fig. 1A and Supplemental Movie 1) on these substrates. Also the maximum distance travelled from the point of origin within 12 hrs was significantly higher for HUVECs and fibroblasts (128 ± 9 µm and 139 ± 19 µm respectively) as compared to ABMSCs (40 ± 4 µm), while the migration range of FBMSCs (91 ± 26 µm) lies in between those values (Figs 1B and S1A). Because cell migration tightly depends on adhesion strength^38^, we next investigated the effect of varying fibronectin density. ABMSC migration velocity remained low on substrates coated within the range of 0–80 μg/ml bovine or human fibronectin (Fig. 1C). To investigate if the age of donors of ABMSC might affect the migration capacity of the cells, we separated the migration data on 20 μg/ml into donor age groups and found no clear correlation between donor-age and migration velocity of MSCs (Fig. S1B).

To establish whether the slow migratory behaviour of MSCs is retained in other media, that are commonly used to culture MSC for clinical therapy^39,40^, we investigated MSC migration in medium containing human derived platelet lysate (PL). These experiments demonstrate that the migration velocity of MSCs is similar in medium supplemented with PL compared to medium supplemented with FCS (Fig. 1D). Integrin-based focal adhesions and their regulation by the actin cytoskeleton are crucial for cell adhesion and migration^41^. To explore whether integrin-dependent adhesions are formed in MSCs, we analysed focal adhesions and the actin cytoskeleton by immunofluorescence staining of ABMSCs, FBMSCs, HUVECs and fibroblasts. All primary cell types stained positive for paxillin-pY118, a marker of integrin-signalling and F-actin-connected focal adhesions, indicating that the basic adhesion complexes needed for migration are formed in MSCs (Fig. 1E). Together, these data indicate that migration of MSCs on fibronectin substrates is inefficient in comparison to related primary human cell types.

### ABMSC migration through confined pores is limited by the nucleus

**Figure 2:**
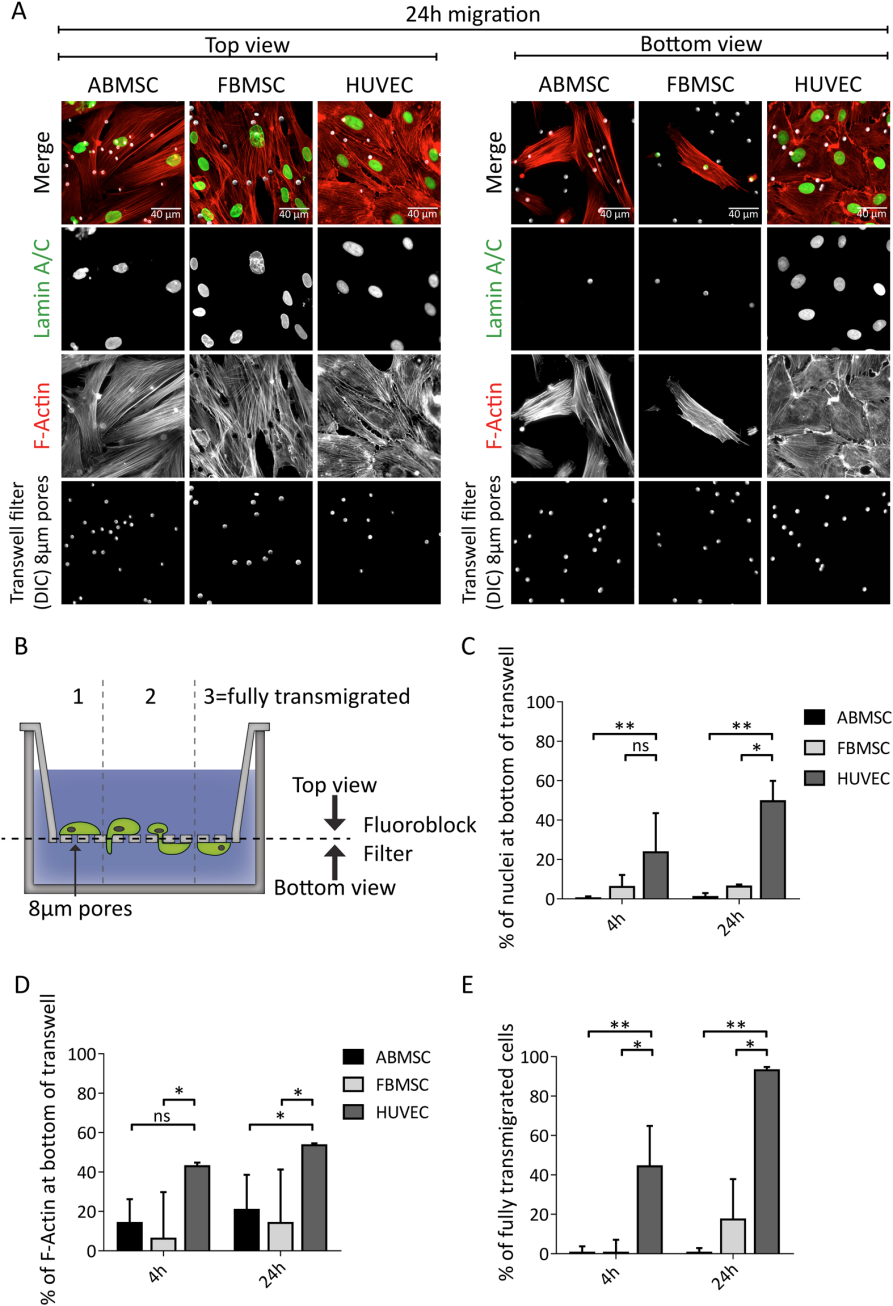
Transwell migration experiments of ABMSC, FBMSC and HUVEC in response to serum. (**A**) Representative widefield immunofluorescence (IF) images of ABMSC, FBMSC and HUVEC at the top (left panel) and bottom (right panel) of a Transwell filter. Cells were allowed to migrate for 24 hours and were subsequently fixed and stained for Lamin A/C (green), F-actin (red). Pore structures (8 µm) were visualized by DIC imaging. Scalebar: 40 μm. (**B**) Schematic overview of Transwell experiments. During transmigration, different migratory stages can be distinguished. Stage 1: cells on top of the Transwell filter that have not started transmigration. Stage 2: cells on top of the Transwell filter that form protrusions through the filter pores and initiate transmigration. Stage 3: cells that have completed transmigration through the pores. (**C**) Quantification of the percentage of transmigrated ABMSC, FBMSC and HUVEC after 4 and 24 hours migration. Percentage is determined from IF tilescan widefield images stained for Dapi and Lamin A/C from both top and bottom comprising the total transwell area. Median ± range. n = 3 independent experiments using cells from 3 different donors. **p < 0.01 (Kruskal-Wallis, multiple comparisons uncorrected Dunn’s test). (**D**) Quantification of total F-actin at the bottom of the Transwell. F-actin surface area coverage at top and bottom of the Transwell filters was determined from IF tilescan widefield images comprising the total transwell area. Cell-body migration (F-actin at Transwell bottom (stage2 + 3) was calculated as a percentage of total F-Actin (all stages combined). Median ± range. n = 3 independent experiments using cells from 3 different donors. ns = non-significant, *p < 0.05 (Kruskal-Wallis, multiple comparisons uncorrected Dunn’s test). (**E**) Quantification of the percentage of protruding cells that are fully transmigrated. Widefield images from the bottom of the filter were manually screened for presence of a nucleus (Lamin A/C) and presence of cell-body (F-Actin). Number of fully transmigrated cells (stage 3) was calculated as percentage of total protruding cells (stage 2 + 3). Median ± range. n ≥ 3 independent experiments including 30 cells each. *p < 0.05, **p < 0.01 (Kruskal-Wallis, multiple comparisons uncorrected Dunn’s test).

To investigate the migration capacity of ABMSCs through spatially restricted environments that occur in tissue^42^, we next used fibronectin-coated transwells to monitor directed cell migration from upper wells towards bottom compartments. Similar to MSCs entrapped in the pulmonary vasculature, migration of MSCs requires cell body and nuclear deformation in this experimental setup. The transwells contained pores with a diameter of 8 µm, close to the size of the smallest capillaries present in the lung vasculature^20^, which is the approximate limiting diameter before physical cell arrest^42^. Serum was used as chemoattractant in the bottom compartment. ABMSC, FBMSC and HUVECs were plated in the top chamber of transwells and fixed for immunostainings of F-actin and the nucleus (anti-Lamin A/C). Fluoroblock inserts were used to separate non-migrated cells (top-filter) from migrated cells (bottom-filter) by microscopy. Imaging of the filter bottoms showed that only few ABMSCs and FBMSCs have passed through the transwell filters after 24 hours, whereas HUVECs readily transmigrated through the confined pores during this time window (right panel, Fig. 2A). Moreover, even though part of the MSC cytoplasm passed through the transwell pores, the corresponding nuclei remained above the filter (Fig. 2A). To analyse these results 3 different stages of migration were defined (Fig. 2B): Stage 1 represents non migrated cells. Stage 2 represents cells that displayed protrusions and large cell body migration through the transwell pores while the nucleus remained behind. Stage 3 represents the completely transmigrated cells, which migrated through the filter membrane with both the cell body and nucleus. To determine the percentage of fully transmigrated cells we quantified the number of cell nuclei at the filter bottom and top after 4 and 24 hours migration. These results indeed show a significantly lower percentage of transmigrating MSCs compared to HUVECs (Fig. 2C). As MSCs do initiate transmigration, as indicated by the presence of F-actin at the filter bottom, we next determined the division of cytoskeletal compartmentalization of cells between top and bottom chambers. Total F-actin levels at both filter sides were captured by imaging the entire transwell surface (Fig. S2A). The ratio between F-actin levels at the bottom and the top of the filter shows that migration is initiated in a significant fraction of ABMSCs (16%) and FBMSC (14%), although HUVECs were at least twice as efficient (41%) (Fig. 2D). We next determined the percentage of fully transmigrated cells (stage 3) as a percentage of the protruding cell subpopulation (only stage 2 and 3 cells). Strikingly, no protruding ABMSCs and only 25% of the protruding FBMSCs have fully transmigrated within 24 hours (Fig. 2E). To ensure that these results were not only caused by slower migration kinetics of ABMSCs and FBMSCs, the experiments were extended to 48 hours and 7 days. Even after 48 hours less than 10% of ABMSCs were fully migrated, whereas at these time points a significant portion of the FBMSCs transmigrated (stage 3, Fig. S2B,C). In conclusion our results clearly indicate that MSCs, and ABMSCs in particular, are inefficient in migrating through confined spaces, which correlates with an apparent inability to squeeze through the 8 µm pores (Supplemental Movie 2).

### The nuclear envelope of ABMSCs is irregularly shaped

**Figure 3:**
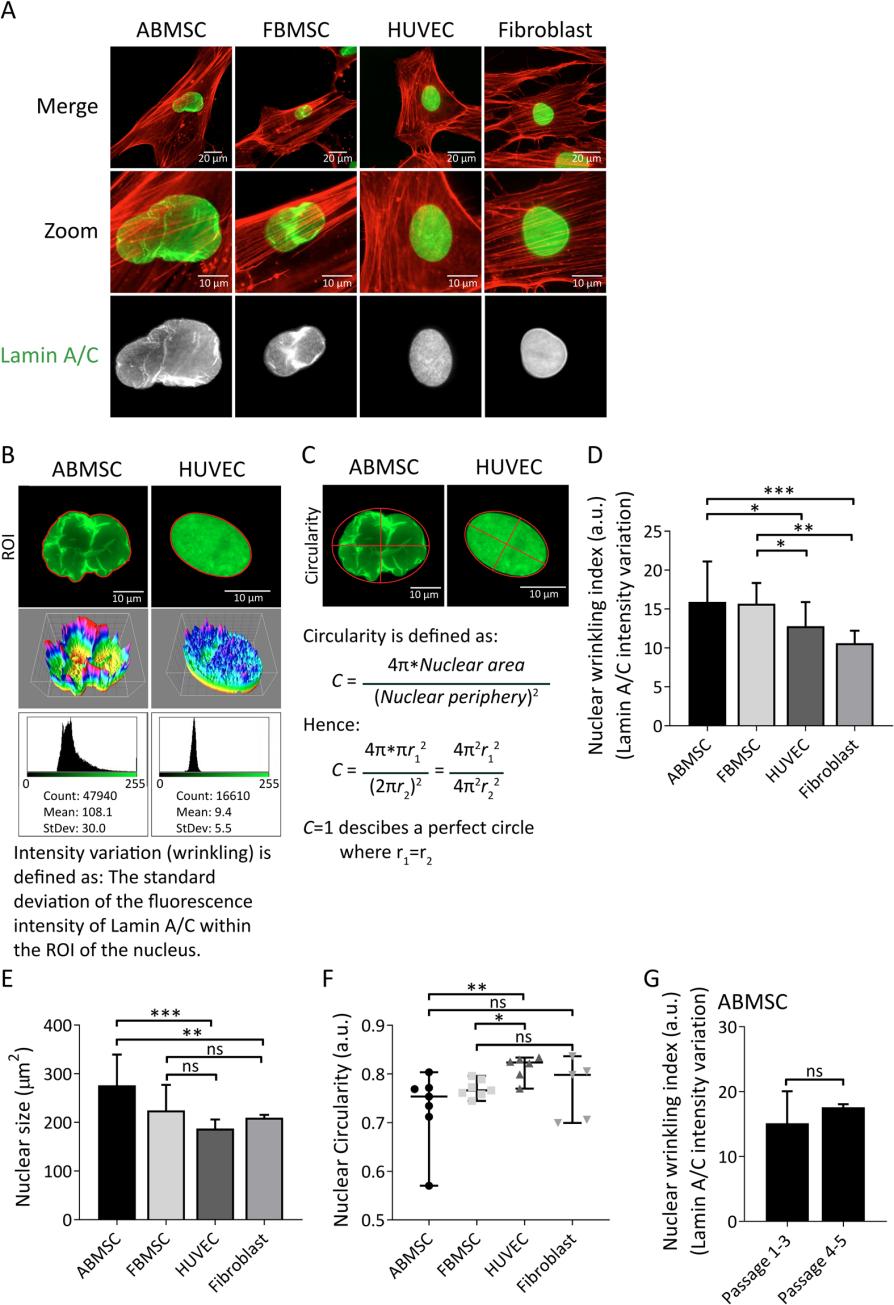
Nuclear characteristics of ABMSC compared to other primary mesodermal-derived adherent cells. (**A**) Representative widefield IF images of ABMSC, FBMSC, HUVEC and fibroblasts stained for Lamin A/C (green) and F-actin (red). Magnified images from regions of interest (zoom) show detailed structures of the nuclear envelope containing Lamin A/C. Scalebars: 20 μm and 10 μm. (**B**) Quantification method of nuclear intensity variations (wrinkling) based on Lamin A/C IF imaging in arbitrary units (a.u.). Regions of interest (ROI) were drawn in ImageJ based on Lamin A/C signal, and the standard deviation of the fluorescence intensities within the ROI was determined and normalized to the mean fluorescence intensity. Two examples are shown to emphasize the differences in morphology. 3D projections visualize the intensity variations within nuclei. (**C**) Quantification method of nuclear circularity, based on Lamin A/C IF imaging. Circularity between different nuclei can be compared by calculating the ratio between nuclear periphery and nuclear surface area. For a perfect circle, these ratio’s result in equal nuclear radii (r_1_ = r_2_). (**D**) Quantification of nuclear wrinkling in different mesodermal-derived adherent cells, based on Lamin A/C IF widefield-images. Intensity variation of Lamin A/C in a.u. was determined with ImageJ. Median ± range. n ≥ 5 independent experiments including 30 cells per experiment and per cell type. *p < 0.05, **p < 0.01, ***p < 0.001 (Kruskal-Wallis, multiple comparisons uncorrected Dunn’s test). (**E**,**F**) Quantification of the nuclear surface area and circularity of ABMSC compared to FBMSC, HUVEC and fibroblasts based on Lamin A/C stainings in widefield images. The surface area in µm^2^ was determined with ImageJ. Median ± range. n ≥ 5 independent experiments including 30 cells per experiment and per cell type. ns = non-significant, *p < 0.05, **p < 0.01, ***p < 0.001 (Kruskal-Wallis, multiple comparisons uncorrected Dunn’s test). (**G**) Intensity variation of Lamin A/C (nuclear wrinkling) in early (p1-3) and late (p4-5) passage ABMSCs. Median ± range. n = 3 experiments, including 3 different donors for each passage condition ns = non-significant (Mann-Whitney test).

Cell migration is determined by proper positioning, shape adaptation and cytoskeletal anchoring of the nucleus^12,27–29,42^. Because the transmigration of MSCs through pores was limited by their nuclei, we next investigated nuclear organization. The nuclear membrane consists of an outer and an inner membrane and the inner nuclear membrane is tethered to a network of intermediate filaments^27,43,44^. This network is predominantly composed of nuclear lamina constituents Lamin A/C (LMNA gene, Lamin A and truncated splice variant Lamin C) and Lamin B. Intriguingly, immunofluorescence stainings for Lamin A/C revealed that the nuclear lamina of MSCs are irregularly organized, in contrast to adhering HUVECs or fibroblasts, which contain a consistent oval nucleus and homogenous Lamin A/C distribution (Fig. 3A). The wrinkling of nuclei in MSC nuclei was independent of the fixation method for imaging or cell culture media (Supplemental Fig. S3A). Comparable examples of irregular folded, or wrinkled, nuclear lamina have been previously described by others in various cell types^32,45,46^. To analyse nuclear wrinkling we determined the Lamin A/C intensity variation (Fig. 3B), circularity and size of nuclei (Fig. 3C) of the various cell types. These quantifications showed that the nuclear envelope of MSCs were significantly more wrinkled than the nuclei of HUVECs or fibroblasts (Fig. 3D). In addition, ABMSC nuclei were larger and less circular compared to HUVEC or fibroblasts (Fig. 3E,F). To investigate whether the observed wrinkling of MSC nuclei arises during culture expansion, we compared the intensity variation between early (p. 1–3) and late passage (p. 4–5) ABMSCs. These experiments show that the high Lamin A/C intensity variation was present in ABMSCs of all passages and from multiple donors, suggesting that nuclear wrinkling is an intrinsic property of the cells (Figs 3G and S3B,C). Our data thus indicate that the nuclei of MSCs are irregularly shaped compared to related cell types.

### Protrusion formation during transmigration of ABMSC is controlled by Lamin A/C expression levels

**Figure 4:**
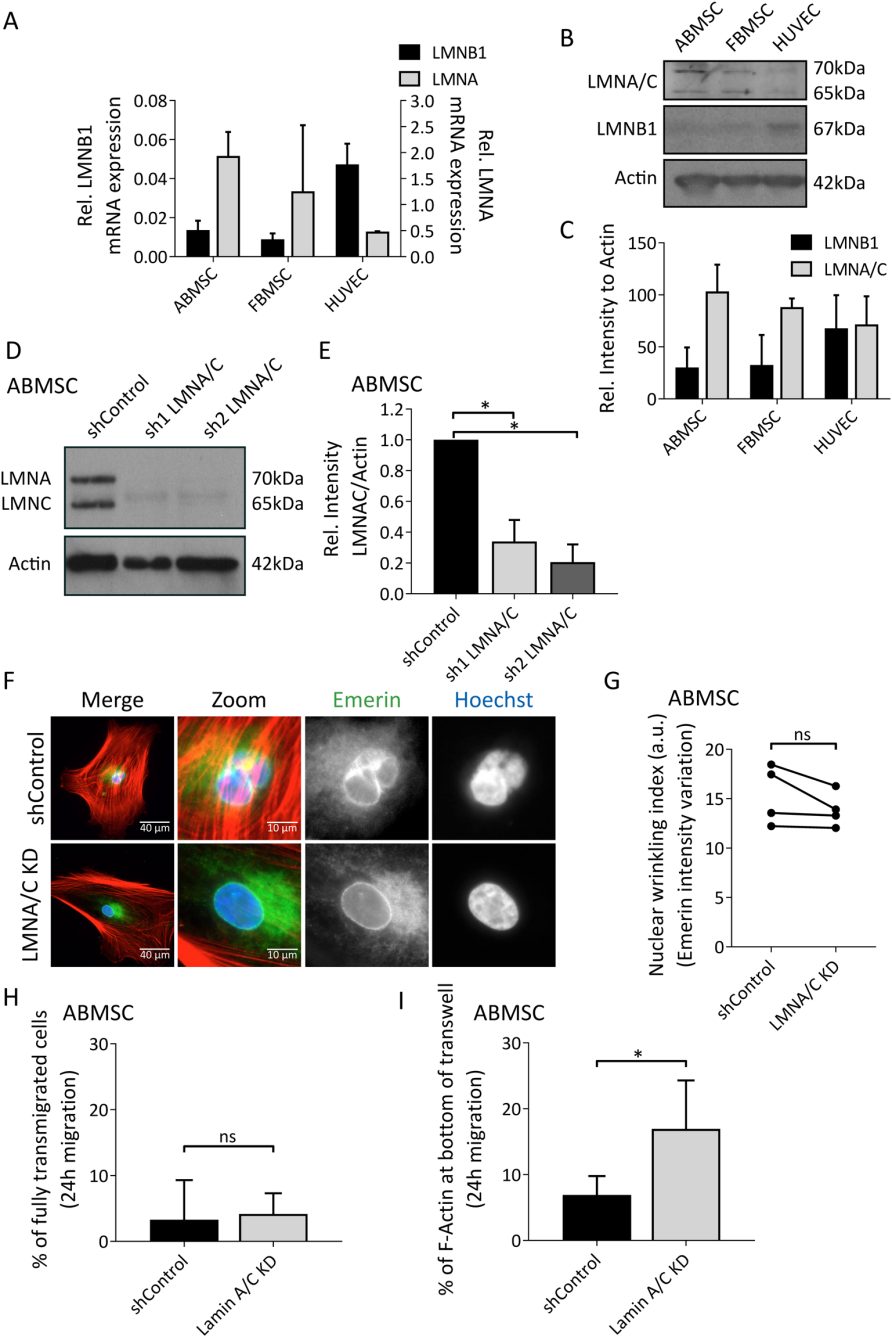
Transmigratory potential of Lamin A/C-depleted ABMSCs. (**A**) LMNB1 (left y-axis) and LMNA (right y-axis) mRNA expression levels in ABMSC, FBMSC and HUVEC relative to Histone Family member 3 A (H3F3A) expressed as 2^−∆Ct^, determined by qRT-PCR. Median ± range. n = 3 independent experiments. *p < 0.05, (Kruskal-Wallis, multiple comparisons uncorrected Dunn’s test). (**B**,**C**) Western blot analysis of Lamin A/C, Lamin B1 and actin (loading control) in lysates of ABMSC, FBMSC and HUVEC. (**B**) Images are cropped scans of blots, corresponding whole Western blot scans are shown in Supplemental Fig. S7A. (**C**) Quantification of Lamin A/C and Lamin B1 protein levels in lysates of ABMSC, FBMSC and HUVEC based on Western blot, analysed with ImageJ. Median ± range. n = 3 independent experiments including cells from 3 different donors. **(**Kruskal-Wallis, multiple comparisons uncorrected Dunn’s test). (**D**,**E**) Western blot analysis of Lamin A/C and actin (loading control) in lysates of ABMSC transduced with shRNA Control or shRNA1,2 targeting Lamin A/C. (**D**) Images are cropped scans of blots, corresponding whole Western blot scans are shown in Supplemental Fig. S7B. (**E**) Quantification of Lamin A/C protein levels in lysates of ABMSC transduced with shRNA Control or shRNA1,2 Lamin A/C based on Western blot, analysed with ImageJ. Mean ± s.e.m. n = 3 independent experiments including cells from 3 different donors. *p < 0.05, (one-sample t-test). (**F**) Widefield IF image of in control and Lamin A/C knockdown ABMSC, stained for F-actin (red), Emerin (inner nuclear membrane protein, green) and Hoechst (blue). Magnified images show the nucleus. Scalebar: 40 μm and 10 μm. (**G**) Quantification of nuclear wrinkling in control and Lamin A/C knockdown ABMSC, based on Emerin IF widefield images. Intensity variation of Emerin in a.u. was determined with ImageJ. n = 4 independent experiments including over 30 cells per condition and cells derived from 4 different donors. (Kruskal-Wallis, multiple comparisons uncorrected Dunn’s test). (**H**) Quantification of fully transmigrated control and Lamin A/C knockdown ABMSC (pool of shRNA1 and 2) after 24 hours migration. Widefield images from the bottom of the filter were manually screened for presence of a nucleus (Hoechst) and presence of cell-body (F-actin). Number of fully transmigrated cells (stage 3) was calculated as percentage of total protruding cells (stage 2 + 3). Median ± range. n = 4 independent experiments including ≥30 cells per condition and cells derived from 4 different donors. ns = non-significant (Mann-Whitney test). (**I**) Quantification of total F-actin at the bottom of the Transwell for control and Lamin A/C knockdown ABMSC (pool of shRNA1 and 2) after 24 hours migration. F-actin surface area coverage at top and bottom of the Transwell filters was determined from tilescan widefield images covering the entire transwell area. Cell-body migration (F-actin at Transwell bottom (stage 2 + 3)) was calculated as a percentage of total F-actin (all stages combined). Median ± range. n = 4 independent experiments including cells derived from 4 different donors. *p < 0.05 (Mann-Whitney test).

Nuclear envelope organization tightly depends on the ratio of Lamin A/C and Lamin B1 and can greatly influence the migratory potential of cells^30^. Quantitative reverse transcriptase PCR (qRT-PCR) indicate that Lamin A mRNA levels are relatively high, and lamin B1 low, in ABMSCs and FBMSCs compared to HUVECs (Fig. 4A). Westernblot analysis of Lamin A/C and Lamin B1 protein levels in ABMSC, FBMSC and HUVEC confirm similar differences on protein levels (Fig. 4B,C). Next we performed knockdowns of Lamin A/C though lentiviral expression of short hairpin RNAs (shRNAs) in ABMSCs. Two separate shRNAs targeting expression of the *LMNA* gene (encoding for Lamin A/C) induced a robust knockdown of protein expression (Fig. 4D,E). Westernblot analysis in lysates of Lamin A/C knockdown cells showed that Lamin B1 levels were unaltered (Supplemental Fig. S4B). Analysis of the nuclei in Lamin A/C knockdowns showed no clear reduction of nuclear lamina wrinkling (Fig. 4F,G; intensity variation was based on immunofluorescence (IF) stainings of the nuclear membrane protein Emerin). Next we compared the migration capacity of shControl and shLamin A/C cells through transwells and find that although complete transmigration was not achieved (Fig. 4H), a significant increase in MSC protrusions was induced by silencing expression of Lamin A/C (Figs 4I and S4A). This indicates that reducing expression of Lamin A/C enhances ABMSC protrusive activity through transwell pores.

### Lung retention of injected MSCs is controlled by Lamin A/C expression levels

**Figure 5:**
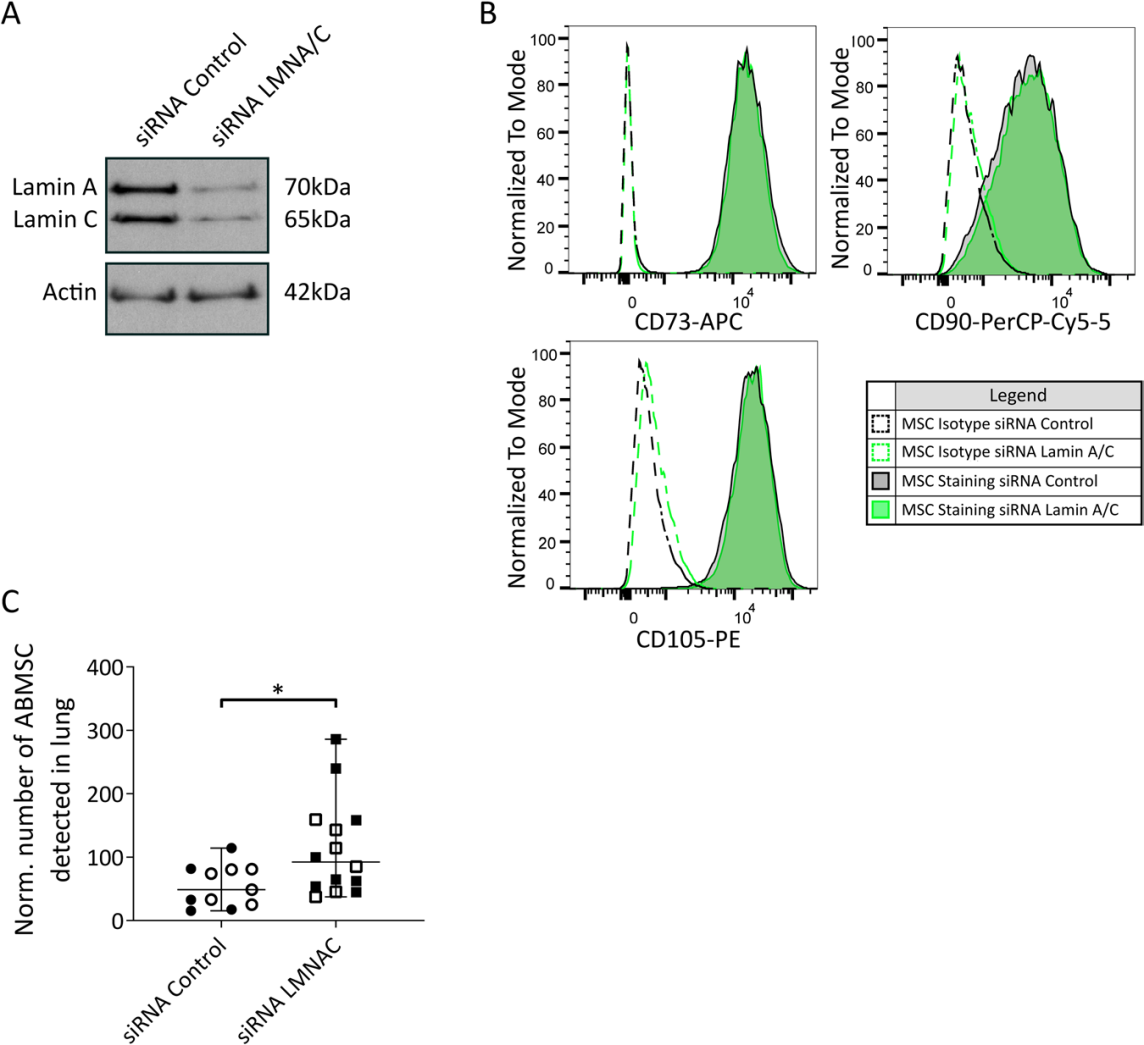
Biodistribution of intravenously injected Lamin A/C-silenced ABMSCs. (**A**) Western blot analysis of Lamin A/C and actin (loading control) in lysates of ABMSC transfected with control or siRNA’s against Lamin A/C. Images are cropped scans of blots, corresponding whole Western blot scans are shown in Supplemental Fig. S7D. (**B**) FACS analysis for the presence of CD73, CD90 and CD105 respectively on ABMSC transfected with siRNA’s control (grey plot) or siRNA’s targeting Lamin A/C (green plot). Dotted graphs display the isotype staining, solid lines display antibody staining. (**C**) Accumulation of siRNA control and Lamin A/C ABMSC in the lung 14 hours after intravenous injection in C57BL/6JRj mice. ABMSC biodistribution was analysed by FACS of the lung tissue. Graph shows the number of detected ABMSCs relative to the total number of isolated cells. Data is normalized to the average number of viable ABMSCs in each experiment. Total siControl ABMSC-injected mice: 11, Total siRNA LMNA/C ABMSC-injected mice: 14, n = 2 independent experiments. Experiment 1: open symbols, Experiment 2: closed symbols. Median ± range *p < 0.05, (Mann-Whitney test).

Because the previous data indicate that reducing expression of Lamin A/C promotes protrusive activity of MSCs *in vitro* and thereby might change the homing of administered MSC *in vivo*, we next investigated the tissue retention of ABSMCs after systemic infusion in mice. Human ABMSC passage 3–4 were transfected with control siRNA or Lamin A/C siRNA and knockdown efficiency was determined after 48 hours (Fig. 5A). Fluorescence activated cell sorting (FACS) analysis showed that prior to injection, and according to the ISCT guidelines^1^, over 95% of the ABMSCs were positive for the human MSC markers CD105, CD90 and CD73, and that Lamin A/C knockdown did not affect the expression of those markers (Fig. 5B). C57BL/6 mice were subsequently intravenously injected with control or Lamin A/C knockdown human ABMSCs (200,000 cells per mouse; corresponding to 10*10^6^ cells/kg, which is 5–10 times higher than in clinical settings^39,40,47^ and lower than in reported *in vivo* studies^22,25,48^). 14 hours after injection animals were sacrificed and different tissues including lung, liver, spleen and blood were analysed for the number of ABMSC residing in tissue using FACS. Viable human ABMSC were detected with a live dead marker and antibodies against human CD105, CD90 and CD73. Tissues of PBS-injected mice served as a negative control. The FACS gating strategy to detect viable ABMSCs in tissue homogenates is provided in Supplemental Fig. S5. These experiments demonstrate the presence of viable human ABMSC in the lungs, but they are absent in blood, spleen and liver tissues (Supplemental Fig. S6). Interestingly, our data show a subtle increase of viable siLaminA/C ABMSC in lung tissues compared to administered siControl ABMSCs (Fig. 5C). Taken together, these data show that reducing Lamin A/C levels restores protrusive activity *in vitro* and leads to enhanced tissue retention in the lung.

## Discussion

Mesenchymal stromal cells have long been studied for their therapeutic effect in a wide range of clinical applications. While Phase II and III clinical trials are ongoing to proof the therapeutic efficiency of MSCs, the localization and faith of MSCs after injection in humans remains unclear to date^49,50^. Tracing MSCs after injection appears to be difficult because of the heterogeneity of the cell population and possibly apoptosis and clearance of the administered cells^51,52^. There is however consensus that migration of MSCs towards tissues after intravenous administration is inefficient^10,18^. Therefore, identifying novel approaches to improve migration and homing of MSC to generate more effective MSC-based therapies are widely being studied^10,12,19,28,29^. Here, we show that MSC migration capacity *in vitro* is intrinsically low compared to other primary mesodermal derived adhering cells (Fig. 1A,B).

Yet, MSC express important regulators of migration and can respond to migratory stimuli^33^. For example, human mesenchymal stromal cells that are stimulated with the chemoattractant SDF-1α show enhanced migration and enhanced focal adhesion turnover *in vitro*^33,53^. In addition MSCs, like other primary cells, form integrin-based adhesions (Fig. 1E). Potential differences between the investigated cell types in integrin signaling might contribute to the limited migration capacity of MSCs, which warrants further investigation. Here, we focussed on the role of the nuclear lamina in MSC protrusive activity *in vitro* and in tissue distribution after intravenous injection in mice. ABMSC contain a high Lamin A/C to Lamin B1 ratio, which likely promotes rigidity of the nucleus and may render MSC nuclei more prone to wrinkling. We further show that the MSC nucleus is a limiting factor for protrusive activity through transmigration pores. Possibly, the intrinsic MSC-specific nuclear properties contribute to substrate stiffness-dependent stem cell differentiation^54–56^.

### MSC migration through confined microenvironments

The lung vasculature forms a critical hurdle for MSC biodistribution and survival after administration. To gain insight in the process of MSCs migration through such spatially restricted environments we performed transwell experiments with pore sizes similar to capillaries in the lung, demonstrating that MSC migration in confined microenvironments is also limited compared to HUVEC (Fig. 2C). While the F-actin of MSC can readily protrude the narrow structures in response to chemoattractants, the MSC nucleus stays behind and prevents full transmigration (Fig. 2D,E). It should be noted that ABMSC cell body and nuclear size are slightly larger compared to HUVEC (Fig. 3E), which might partially affect transmigration speed. Cell-body size, nuclear size, shape or composition can vary with different cell culture protocols or tissue origin of the MSC. For example, the expansion of MSC in culture media supplemented with platelet lysate instead of FCS^57^, or the culture of MSC in suspension^58^, can result in a reduction of cell size. Possibly, the migration of MSC may be further enhanced through optimization of the current (clinical) cell culture protocols in use.

Of interest, cellular sensing of mechanical cues in 3D environments is mainly regulated through nuclear signalling. Enucleated cells for example show a highly diminished migration capacity through confined spaces even though their cell body is smaller compared to intact cells, and despite the fact that these cells are still able to polarize and migrate in 2D^59^. The fact that ABMSCs are slow migratory cells, might also partially contribute to their inability to pass through the transwell pores, although even after prolonging the migration assays to 48 hours or 7 days, the majority of ABMSCs remained in an intermediate migration phase where the nucleus did not pass the membrane pores (Fig. S2). It should be noted that the actual size and shape of MSC and their nuclei at the moment of intravenous administration is currently not known and of interest for future studies aiming at a better understanding of the biodistribution of MSCs in therapy. Furthermore, the notion that isolated MSCs have an intrinsic low migration capacity and wrinkled nucleus suggests that these cellular properties are not hampering their normal biological function in tissue of origin.

### Composition of the nuclear lamina affects nuclear shape and cell behaviour

Stiffening of the MSC nucleus during differentiation, or as a response to matrix rigidity, can be established through restructuring of the nuclear lamina and results in increased resistance to mechanical deformation^54,55,60^. Migration of cells through spatially restricted environments sets a high mechanical challenge on the cell’s nucleus^30^. This can lead to chromatin distortion, damage, DNA breakage and nuclear rupture. However, migration-induced damage can to some extend be reversed by repair machineries, depending on the duration and severity of nuclear damage^61,62^. Different genetic mutations in the Lamin A/C gene are known to cause morphological changes to the nucleus and are linked to diseases called laminopathies^63^. Examples are Emery-Dreifuss-muscular dystrophy 2/3^64^ and Hutchinson Gilford progeria syndrome^65^. Interestingly, the morphology of nuclei from ABMSC is very similar compared to cell-nuclei from Hutchinson Gilford progeria patients^66^. Moreover, we found that the ratio between the expression levels of nuclear lamina genes and proteins *LMNA* and *LMNB1* are relatively high in MSC compared to HUVEC (Fig. 4A–C). This shift in lamina composition, and consequent effects on nuclear flexibility, likely explains the reduced transmigration of MSCs through small pores. Of note, the potential influence of another B type lamin, Lamin B2, remains to be investigated. Importantly, we found that silencing the expression of Lamin A/C improved the protrusive activity of MSCs in transmigration assays. While the precise molecular mechanism behind this effect is still unknown, it is possible that reduced Lamin A/C levels result in an altered coupling between the nucleus and cytoskeleton allowing MSCs to respond more readily to chemotactic signalling^67^. Our data show that both fetal and adult MSCs (age varying from 25 to 78 years) have reduced migration capacity and display similar differences in nuclear shape, strongly indicating that this is a cell type specific property. Yet, at this point, it cannot be fully excluded that donor age or culture conditions contribute to the reduced migration properties as well. Therefore larger-scale studies including MSC isolations from various age groups might bring further insights.

### *In vivo* administration of MSCs

Previous transplantation studies of hearts and lungs in humans have shown that tissue resident MSCs are predominantly (>95%) from donor origin, indicating the low infiltration of recipient MSC into the donor tissue^68,69^. In addition, after allogeneic bone marrow transplantations the stromal compartment remains from recipient origin, emphasizing the immobile nature of MSCs^70^. Furthermore, after intravenous administration of MSC in mice, the majority of cells is cleared from the bloodstream within 5 minutes and accumulates in the lungs^49^. We investigated directly whether knocking down expression of Lamin A/C in MSCs affects tissue retention after intravenous injection *in vivo*. In the mouse model we administered 10*10^6^ MSC/kg, a dosage that is in between the common dosages used in human clinical settings (1–2*10^6^ cell/kg^39,40,47^) and murine studies (25–250 * 10^6^ cells/kg^22,25,48^). It should be noted that the concentration of suspended cells was higher compared to human clinical settings due to the limited volume that can be administered in a mice, which might have potential effects on the *in vivo* migration or survival of MSC after administration. The results indicate that Lamin A/C knockdown promotes the accumulation of MSCs in the lung (Fig. 5C). We assume that this effect is caused by: i) increased tissue invasion of the MSCs due to increased protrusive activity and/or ii) due to increased compliance (e.g. more flexible or less anchored nucleus) within the narrow vascular environment leading to prolonged cell survival. In general, the lung vasculature constitutes a barrier for MSC migration and administration of vasodilators might further promote the distribution of MSCs towards distant organs after intravenous injection^24^. In contrast to other studies^71^, we did not detect administered MSCs in blood, liver or spleen after 14 hours. A difference which might be caused by differences in detection approaches that are often based on DNA, mRNA or detection of radioactive signals and might not reflect the number of living cells.

### Clinical approaches for MSCs as cellular therapy

For clinical purposes, MSCs are cultured up to passage 4 or 5, although mostly passage 1 or 2 cells are administered^72^. In our studies we included data of MSCs up to passage 5 to avoid effects of long term cultures. Early passage MSCs are also preferred for cell therapy to prevent spontaneous alterations induced by culture. Moreover, early passage MSCs have proven a higher efficiency in therapies against graft versus host disease^73^. Interestingly, the positive clinical effects achieved through MSC administration do not solely depend on their tissue engraftment. While local effects of engrafted MSCs likely contribute to their therapeutic capacity, also factors that are secreted by MSCs systemically play a role. MSCs entrapped in the lung vasculature become activated and promote secretion of anti-inflammatory proteins such as TSG-6^74^. Moreover, the immunosuppressive effect of MSCs might depend on the host ability to kill MSCs and the apoptotic derivatives might accomplish immunomodulatory effects^48^. This suggests that the mechanism of MSC-induced immunomodulation might be comparable with the mechanism of thrombopoiesis where the shedding of platelets from megakaryocytes requires shear stress from the bloodstream^75,76^. Nuclear lamins are essential regulators of genome organization and gene expression. If the presence of MSC cytoplasm or secretory profile is sufficient to establish immune regulatory effects, one might consider more rigorous methods in the preparation of MSC such as denucleation of the cells to enhance tissue retention. Cytoplasts from fibroblasts are stable for 48 hours and still respond to chemotactic and haptotactic gradients, although migration in 3D and response to matrix rigidity is perturbed^59^. Advantage of this approach is that cytoplasts might pass more easily through the microvasculature and therefore show a longer circulation time. Also, any potential malignant transformation of administered MSCs is avoided by removing nuclear DNA. Obviously, this method would not be suitable for cell-based therapies with a regenerative purpose, such as treatment of osteogenesis imperfecta or cartilage repair, where MSCs need to engraft, expand and differentiate in order to be effective.

### Perspectives

Taken together, we find that MSCs have a particular slow capacity to migrate in 2D as well as in confined 3D microenvironments compared to other cells. These special properties of MSCs depend on differences in nuclear lamina constituents. Protein and mRNA expression analysis shows that the levels of nuclear lamina proteins Lamin A/C are relatively high compared to Lamin B1 in MSCs compared to HUVECs. We show that reducing expression of Lamin A/C promotes protrusive activity of MSCs and enhances MSC retention in the lungs after intravenous administration *in vivo*. These results contribute to a better understanding of the low engraftment and migration capacity of MSCs upon intravenous administration in clinical settings. Our data further suggest that novel to-be-developed approaches that are aimed at modulating the structural properties of the nucleus, might be valuable to improve migration and engraftment of administered MSCs.

## Materials and Methods

### Cells and cell culture

Human adult bone marrow mesenchymal stromal cells (ABMSCs) and fetal bone marrow mesenchymal stromal cells (FBMSC) were isolated and cultured as described previously^33^. In summary, adult bone marrow MSC (BMSC) aspirates were obtained from the sternum of patients undergoing cardiac surgery. Fetal bones were flushed with Iscove’s modified Dulbecco’s medium (IMDM) containing 10% FCS and 1% penicillin-streptomycin. MSC were isolated by density gradient centrifugation (Ficoll-paque, 1.077 g/ml; Cat# 17144003, GE life sciences). The remaining erythrocytes in the cell suspension were lysed using NH_4_Cl for 10 min on ice. Subsequently, cells were rinsed in PBS. 1*10^6^ cells (FBMSC) or 5*10^6^ cells (ABMSC) were seeded per well in a 6-well dish in DMEM containing 10% FCS and 1% penicillin-streptomycin. Collection of fetal tissues for research purposes was approved by the medical ethical review board of the Academic Medical Centre (AMC) (MEC: 03/038). The fetal tissue samples were obtained from the HIS facility of the AMC, Amsterdam. All material has been collected from donors from whom a written informed consent for the use of the material for research purposes had been obtained by the Bloemenhove clinic (Heemstede, The Netherlands). These informed consents are kept together with the medical record of the donor by the clinic. 6 FBMSC independent donors were included in this study. Collection of adult bone marrow aspirates for research purposes, after informed consent, was approved by the medical ethical review board of the AMC (MEC:04/042#04 17 370). Donor age in this study ranges from 25 to 78 years with an average of 62 years of age and include both males and females. A total of 22 ABMSC donors were included in this study. Primary human umbilical vein endothelial cells (HUVEC) were obtained from Lonza and consist of pools from different donors. 4 independent pools of HUVEC were included in this study. Primary human fibroblasts were obtained from skin biopsies of three healthy adolescent donors^77^. All experiments were performed in accordance with relevant guidelines and regulations. All experiments were performed with cultured cells ranging from passage 1–5. Unless specified differently, MSCs and fibroblasts were cultured in Dulbecco’s Modified Eagle’s Medium (DMEM), Cat# 11966025, ThermoFisher, supplemented with 10% FCS and 1% penicillin–streptomycin and HUVECs in BulletKit supplemented endothelial cell growth medium-2 (EGM-2), Cat# CC-3162, Lonza.

### Quantitative reverse transcriptase PCR (qRT-PCR)

RNA was extracted with a RNeasy Micro Kit (Cat# 74004, Qiagen) and concentration was measured with the Nanodrop bioanalyzer. cDNA was synthesized with random hexamers and M-MLV Reverse Transcriptase (Cat# 28025-021, Invitrogen). Quantitative real-time PCR was performed on a StepOne Plus (Applied Biosystems, Warrington, UK). SYBR™ Green PCR Master Mix (Cat# 4309155, ThermoFisher), was used for amplifications. The mRNA expression was calculated as fold-change relative to Histone fam 3A mRNA expression (2^−∆Ct^). 5′-3′ primer sequences were as follows: Histone fam 3a forward AAACTTCCCTTCCAGCGTCT and reverse GTCTTCAAAAAGGCCAACCA; Lamin B1 forward TGGAGTGGTTGTTGAGGAAG and reverse TCTATTGGATGCTATTGGGGTT; Lamin A/C forward TGGATGAGGAGGGCAAGTTT and reverse CGGTAAGTCAGCAAGGGA.

### Western immunoblotting (WB)

Cell lysates were prepared in reduced sample buffer and analysed by standard western immunoblotting and enhanced chemiluminescence detection. Antibodies used for western immunoblotting were: Mouse monoclonal Lamin A/C ((636) Cat# sc7292, WB 1:2000) from Santa-Cruz Biotechnology, Rabbit polyclonal Lamin B1 (Cat# PA5-19468, WB: 1:1000) from ThermoFischer Scientific. Mouse monoclonal Actin ((AC-40) Cat# A3853, WB: 1:3000) from Sigma-Aldrich. Secondary antibodies were: Goat anti-mouse HRP (Cat # P044701-2, WB: 1:5000) and Swine anti-rabbit HRP (Cat # P039901-2, WB: 1:5000) from Agilent.

### Image analysis

Image analysis was performed using ImageJ software (National Institutes of Health). For analysis of cell migration, cells where tracked manually using the ImageJ software plugin MTrackJ. For more information on the tracking software see^78^. The centre of the nucleus was used as a reference point. We used the following parameters calculated by the MTrackJ program for our analysis: The velocity: v [µm/minute] and the distance to start: D2S [µm]. The cell velocity [v] is given for every point in the track as follows: The distance from the previous to the current tracking point, divided by the frames time-interval. Subsequently, the average velocity of the entire track was calculated. The distance to start [D2R] is given for every point in the track as follows: Distance from the start (first point) to the current tracking point. The maximal migration range for each cell is defined as the maximal distance on the migration track from the start position. Nuclear shape and morphology were quantified in ImageJ by automated contour determination of the nucleus using the magic wand tool to define the region of interest (ROI) capturing the nucleus. Nuclear wrinkling was defined as the immunofluorescence intensity variation within the ROI. Circularity of the nucleus was determined by the area to periphery ratio where a ratio of 1 represents a perfect circle. Analysis of transmigration (Fig. 2C,D) were based on tilescan images of the entire top and bottom of the transwell filter stained for total F-actin, Lamin A/C and Dapi. Migration of the nuclei (Fig. 2C) was determined based on by Lamin A/C and Dapi stainings as follows: The ImageJ plugin “Find Maxima” was used to count nuclei on top and on bottom of the transwell filters. Migration was determined as the number of nuclei at the bottom expressed as a percentage of total nuclei detected. Migration of the cytoskeleton (Fig. 2D) was determined based on F-actin stainings as follows: Images were thresholded using Otsu method, number of positive pixels on bottom of transwell was determined within the transwell area and expressed as a percentage of total pixels (top and bottom of transwell) stained positive for F-actin.

### 2D migration assays

24 well plates were coated with bovine plasma fibronectin (20 µg/ml) for 30 minutes at 37 °C. Alternative substrate coatings are specified in the figure legends. ABMSC, FBMSC, HUVEC and Fibroblasts were seeded at 2 * 10^3^ cells/cm^2^, adhered for 4 hours and were subsequently imaged for 12 hours at 37 °C, 5% CO_2_. For analysis of cell migration on 2D fibronectin substrates, 4 independent experiments were performed including ABMSC from 4 different donors, FBMSC from 4 different donors, HUVEC from 2 different pools comprising at least 3 different donors each and Fibroblasts from 3 different donors. For each condition (i.e. ABMSC, FBMSC, HUVEC and Fibroblasts) and in every experiment the velocity and migration of at least 30 cells were analysed.

### Transwell assays

8 µm pore size Transwell filters for 24-wells plates contained a light blocking polyethylene terephthalate (PET) membrane (Cat# 351152, Corning) for immunofluorescence (IF) widefield imaging or a clear PET membrane (CAT# 3464, Corning) for confocal imaging. Filters were coated with bovine plasma-derived fibronectin (20 µg/ml) for 30 minutes at 37 °C and washed twice with Iscove’s Modified Dulbecco’s Medium (IMDM) containing 0.5% bovine serum albumin (BSA) or endothelial cell growth basal medium-2 (EBM-2) (Cat# CC-3156, Lonza) containing 0.5% BSA (for MSC or HUVEC respectively). Transwells were placed in a 24-wells plate containing 600 µl of migration medium (MSC: IMDM supplemented with 20% FCS, for HUVEC: EGM-2 supplemented with 10% FCS), 100 µl cell suspension was added the top compartment (MSC: 5*10^4^ cells/ml; HUVEC: 25*10^4^ cells/ml in starvation medium), and cells were allowed to migrate for 4, 24, 48 hours or 7 days. After migration, Transwells were washed once with PBS++ (PBS supplemented with 1 mM CaCl2 and 0.5 mM MgCl2) and fixed in 4% paraformaldehyde (PFA) in PBS++. To proceed for IF stainings, Transwell filters were cut out using a scalpel. After staining Transwells were mounted in Mowiol 4-88/DABCO between two thin coverslips to view both top and bottom of Transwell. All time points 4, 24 h (Fig. 2A,C–E), 48 h and 7 days (Supplemental Fig. S2B, C) included at least 3 independent experiments and included different donors for every experiment.

### Antibodies and reagents

Mouse monoclonal Lamin A/C ((636) Cat# sc7292, IF 1:200, WB 1:2000) from Santa-Cruz Biotechnology, rabbit polyclonal Lamin B1 (Cat# PA5-19468, WB: 1:1000) from ThermoFischer Scientific. Mouse monoclonal Actin ((AC-40) Cat# A3853, WB: 1:3000) from Sigma-Aldrich. Hoechst ((33342), Cat# H1399, IF 1:50), Rabbit polyclonal phospho-Paxillin (pY118, Cat# 44-722 G, IF 1:200), Phalloidin (Texas red 568, Cat# T7471, IF 1:400), secondary antibodies Chicken anti-mouse (AF488, Cat# A21200), Chicken anti-goat (AF488, Cat# A21467), Goat anti-Rabbit (AF633, Cat# A21070) were all diluted 1:200 for IF and obtained from ThermoFisher Scientific. Phalloidin (AF415, Cat# PK-PF415-7-01, IF 1:250) was from PromoKine. Emerin (clone CL0203, Cat# AMAb90562, IF 1:200) was from Atlas Antibodies. Fibronectin was from bovine plasma (Cat# F1141-2MG, Sigma-Aldrich) or human plasma (Cat# F0895-1MG, Sigma-Aldrich), final working concentration 20 µg/ml.

### Immunofluorescence (IF) microscopy

For standard IF staining, cells were cultured on coverslips coated with 20 µg/ml fibronectin for >24 hours and subsequently fixed in 4% paraformaldehyde (PFA) in PBS++ for 10 minutes. Cells were permeabilized with 0.5% Triton X100 in PBS for 5 minutes and blocked for 30 minutes with 2% bovine serum albumin (BSA) in PBS. Primary and secondary antibody stainings were performed in 0.5% BSA in PBS for 45 minutes, and coverslips (#631-0713, ThermoScientific (VWR), diameter: 12 mm, thickness no1: 0.13-0.16 mm) were mounted in Mowiol 4–88/DABCO. IF stained samples were imaged using inverted Widefield microscopes Observer.Z1 and Axiovert 200 M (Zeiss) equipped with 20x and 40x oil objectives (EC Plan-Neofluar 20x/n.a. 0.5 oil and 40x/n.a. 1.3 oil or Plan-Apochromat 40 x /n.a. 1.4 oil). Cell migration was imaged with a 10x and 20x air objective (EC Plan-Neofluar 10 x /n.a. 0.3, EC Plan-Neofluar 20x/n.a. 0.5). Tilescans of transwells were imaged with a 20x air objective (EC Plan-Neofluar 20x/n.a. 0.50). Zeiss Widefield microscope filtersets included: **Dapi**: Filter set 49, #488049-9901-000, Excitation: G 365, Beam splitter: FT 395, Emission: BP 445/50 **GFP**: Filter set 38 HE, #489038-9901-000, Excitation: BP 470/40 (HE), Beam splitter: FT 495 (HE), Emission: BP 525/50 (HE) **RFP**: Filter set 63 HE, #489063-0000-000, Excitation: BP 572/25 (HE), Beam splitter: FT 590 (HE), Emission: BP 629/62 (HE). Software: Zeiss Zen Microscope software, Cameras: Hamamatsu ORCA-R2/C10600-10B, Light sources: Zeis HXP 120 C and Zeis HXP120V Illuminator. IF Z-stacks of ABMSC in transwells were acquired using a TCS SP8 confocal microscope (Leica) equipped with a 40x objective (HC PL APO CS2 40x/n.a. 1.30 oil). Leica TCS SP8 confocal microscope: **Dapi**: Laser line: 405, Intensity: 64%, detector HyD 1 (430–491), Gain: 49, **GFP**: Laser line: 488, Intensity: 3.5%, detector HyD3 (494–557), Gain: 10, **RFP**: Laser line: 561, Intensity: 1.6%, detector HyD3 (654–697), Gain: 30. Images were enhanced for display with an unsharp mask filter and adjusted for brightness/contrast in ImageJ.

### DNA constructs and lentiviral transductions

Transient transfection of constructs was performed with TransIT-LT1 (Mirus Bio) according to the manufacturers’ standard protocol. Lamin A/C expression was knocked down using shRNA expression plasmids based on the RNAi Consortium (TRC) library^79^, validated MISSION TRC1 clones 0000061833 and 0000262764. Both shRNAs induced efficient knockdowns in MSCs and are referred to as shRNA1 LMNAC and shRNA2 LMNAC in the manuscript. Non-targeting shRNA (SHC002; Sigma-Aldrich) was used as a negative control. HEK293T cells were transiently transfected with third-generation packaging constructs and the lentiviral expression vectors. Supernatant containing lentiviral particles was used to transduce MSCs. MSCs were re-plated 24 h prior to transduction at 60% confluency and infected with lentiviral particles for at least 72 h. Subsequently MSCs were subjected to 1 µg/ml puromycin selection for 72 hours prior to analysis.

### Animal Procedures

To comply with animal welfare protocols, MSC were transfected with siRNA’s instead of lentiviral shRNA’s. Human ABMSC passage 3–4 were transfected with either control siRNA or a mix of Lamin A/C siRNAs. siRNAs used were from Sigma-Aldrich: siControl (CAT# siC001-10nmol), siRNA1 LMNAC (CAT# NM_005572, SASI_Hs01_00100729, sequence start 921), siRNA 2 LMNAC (CAT# NM_005572, SASI_Hs02_00339850, sequence start 620). siRNA1 and 2 were mixed in a 1:1 ratio, final concentration of siControl and siRNA mix was 10 μM. For the transfection mix 1.5 μl Dotap (10U/μl, CAT# 11202375001, Sigma-Aldrich) 1.5 μl siRNA and 100 μl Opti-MEM (CAT# 31985070, ThermoFisher) per 1 cm^2^ cell culture surface were mixed and incubated for 10 minutes. Cell culture medium was removed from the cells and transfection mix was added. After 1.5 hour 20 μl IMDM containing 10% FCS was added per cm^2^ culture surface and 2.5 hours later an additional 80 μl IMDM containing 10% FCS was added per cm^2^. Knockdown efficiency was determined 48 hours after transfection and cells were injected the same day. For murine experiments, female C57BL/6 mice were used. The experiment was performed twice, with 6 animals and 8 animals per group respectively. Mice were intravenously injected with either 200,000 control or Lamin A/C knockdown human ABMSC in 150 μl PBS. ABMSC from one donor (first experiment) or a pool of 3 different donors (second experiment) were applied. In addition, for each experiment 2 mice were injected with PBS to serve as a negative control. 14 hours after injection animals were sacrificed and different tissues including lung, liver, spleen and blood were analysed for the presence of ABMSC. All animal experiments were approved by the Experimental Animal Committee of the Netherlands Cancer Institute, according to institutional and national guidelines.

### Murine cell isolation and FACS analysis

Tissues were digested at 37 °C for 30 minutes in 5 ml HBSS buffer (CAT# 24020117, ThermoFisher) containing 2% FCS, 5 mg/ml liberase and 10 mg/ml DNAse (CAT# 5401127001 and CAT# 10104159001, Sigma-Aldrich). All single cell suspensions were filtered through 70μm cell strainers (CAT# 431751, Corning) using a syringe plunger. Cells were washed with PBS containing 2% FCS and 2 mM EDTA and erythrocytes were lysed for 3 minutes at room temperature (155 mM NH_4_Cl, 10 mM KHCO_3_, 1.27 mM EDTA, pH7.4) Cells were washed once more in MACS buffer and total number of isolated cells was determined. ABMSC were stained with antibodies against CD105 (CAT# 560839), CD90 (CAT# 561557), CD73 (CAT# 560847) from BD Biosciences and a live dead marker (CAT# L10119, ThermoFisher). FACS LSR II and LSR Fortessa (BD Biosciences) were used for flow cytometric analysis.

### Statistics

For the *in vivo* experiments, the number of detected ABMSC was calculated as a percentage of total isolated cells. Data from two independent experiments were normalized for the average percentage of detected viable ABMSC in each experiment and then pooled for analysis. Three data points in the control group exceeded the distance from the median with 1.5 times the interquartile range (difference between the 75^th^ and 25^th^ percentile) and were marked as outliers. For all *in vitro* experiments involving single cell measurements, the number of cells analysed are indicated in the figure legends. Single cell measurements were considered technical replicates, and within every independent experiment these measurements are averaged into one single data point. For all statistical analysis in the manuscript, the Mann-Whitney test was used to analyse differences between two experimental groups, for multiple group comparisons Kruskal-Wallis-test followed by an uncorrected Dunn’s test was performed as indicated in the figure legends, P-values of ≤ 0.05 were considered significant.

## Supplementary information

Supplemental Figures and Legends

Migration of ABMSC, FBMSC, HUVEC and fibroblasts.

Z-stack of ABMSC migrating in Transwell assay.

## Data Availability

The datasets generated and analysed during the current study are available from the corresponding author on reasonable request.
