# Supplementary material for: Nuclear shape, protrusive behaviour and in vivo retention of human bone marrow mesenchymal stromal cells is controlled by Lamin-A/C expression
Source: Sci Rep. 2019 Oct 7;9:14401. doi: 10.1038/s41598-019-50955-x (PMC6779744; doi:10.1038/s41598-019-50955-x)
Supplement: Supplementary file 1 — Supplemental Figures and Legends [file 41598_2019_50955_MOESM1_ESM.pdf]

Nuclear shape, protrusive behaviour and in vivo retention of human bone marrow mesenchymal stromal cells is controlled by Lamin A/C expression.

Yvonne L. Dorland[1], Anne S. Cornelissen[2], Carlijn Kuijk[2], Simon Tol[1], Mark Hoogenboezem[1], Jaap D. van Buul[1], Martijn A. Nolte[2], Carlijn Voermans[2#] & Stephan Huveneers[3#]

[1] Department of Molecular and Cellular Hemostasis, Sanquin Research and Landsteiner Laboratory, Academic Medical Center, University of Amsterdam, Amsterdam, The Netherlands.
[2] Department of Hematopoiesis, Sanquin Research and Landsteiner Laboratory, Academic Medical Center, University of Amsterdam, Amsterdam, The Netherlands.
[3] Department of Medical Biochemistry, Academic Medical Center, University of Amsterdam, Amsterdam, the Netherlands.

[#] These authors contributed equally

Short title: Dorland, *et al*.: Nucleus of MSCs limits migratory behaviour

Key words: Mesenchymal stromal cell, nuclear lamina, Lamin A/C, migration, cellular therapy

Correspondence to: Dr. Stephan Huveneers
Amsterdam UMC, University of Amsterdam, Department of Medical Biochemistry,
Meibergdreef 9, 1105AZ, K1-116, Amsterdam, the Netherlands;
E-mail: s.huveneers@amc.uva.nl

Figure S1

A

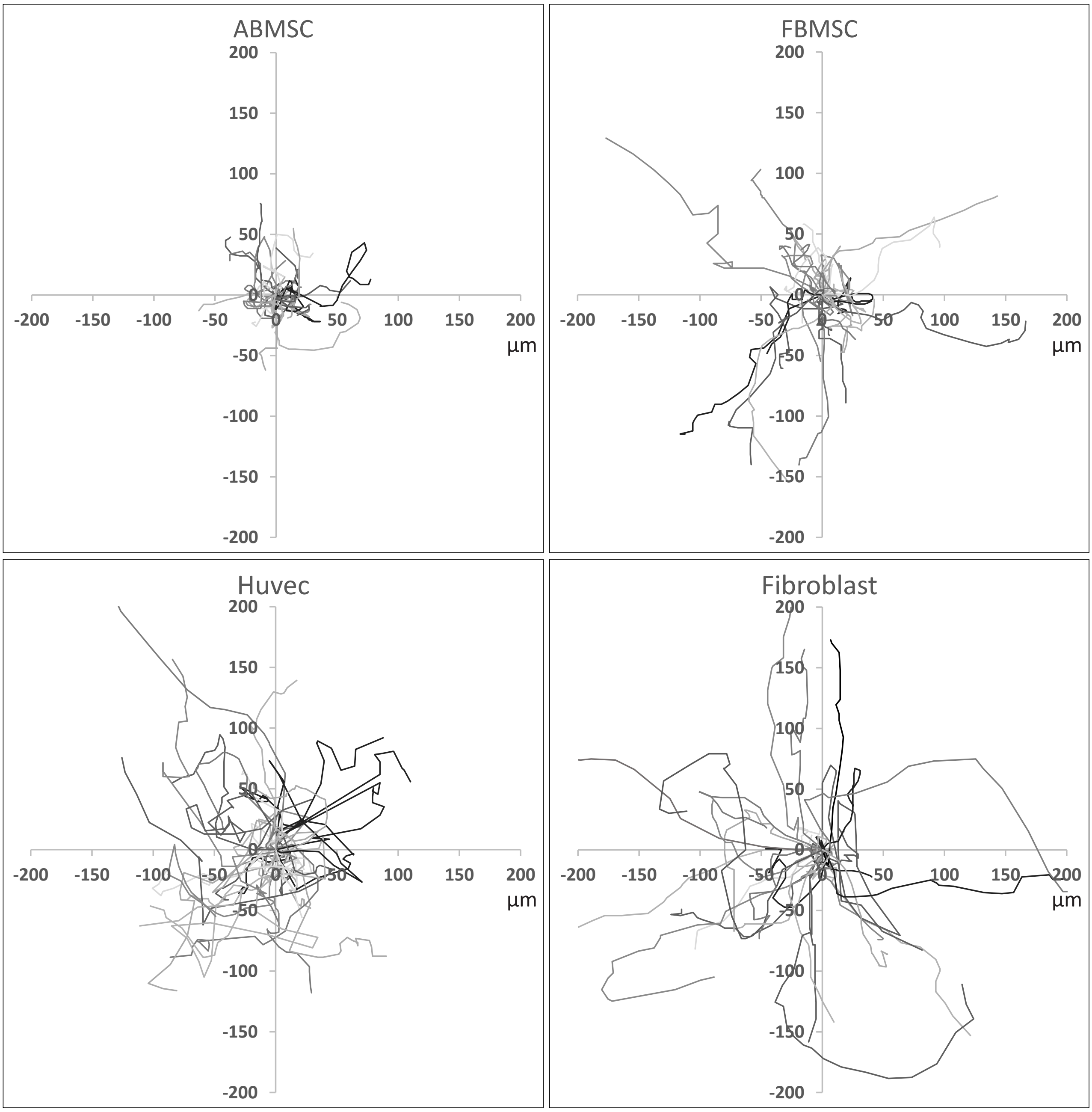
ABMSC
FBMSC
Huvec
Fibroblast
200
150
100
50
0
-50
-100
-150
-200
µm

B

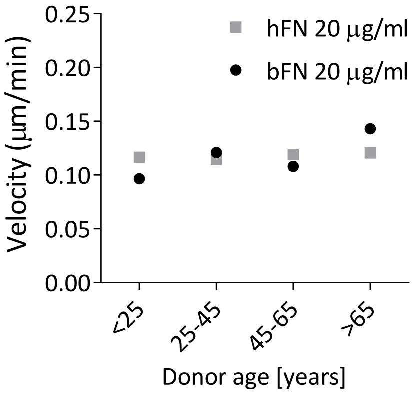
hFN 20 µg/ml
bFN 20 µg/ml
Velocity (µm/min)
0.25
0.20
0.15
0.10
0.05
0.00
<25
25-45
45-65
>65
Donor age [years]

Figure S2

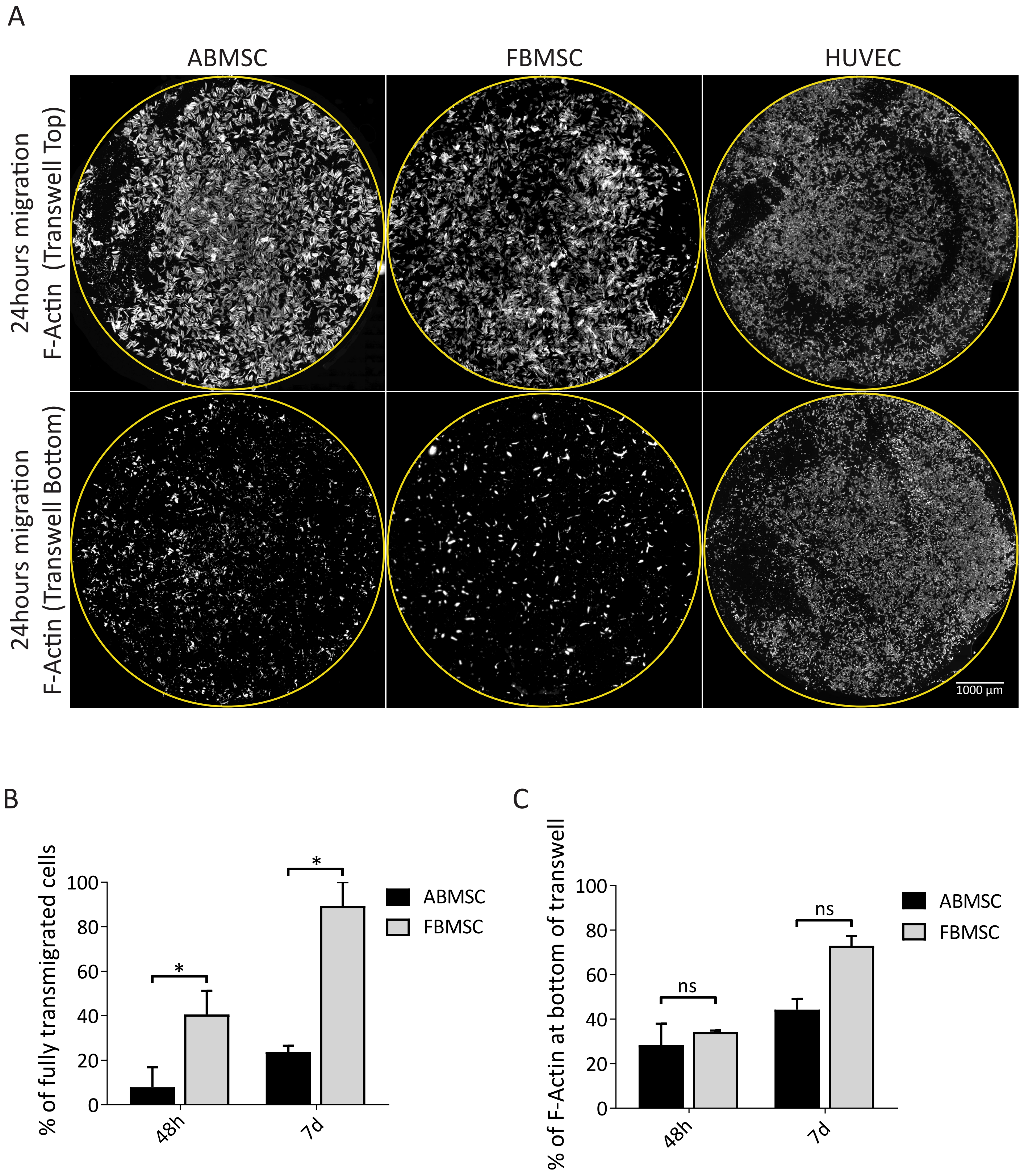
A
ABMSC
FBMSC
HUVEC
24hours migration F-Actin (Transwell Top)
24hours migration F-Actin (Transwell Bottom)
1000 µm
B
% of fully transmigrated cells
100
80
60
40
20
0
48h
7d
*
*
ABMSC
FBMSC
C
% of F-Actin at bottom of transwell
100
80
60
40
20
0
48h
7d
ns
ns
ABMSC
FBMSC

Figure S3

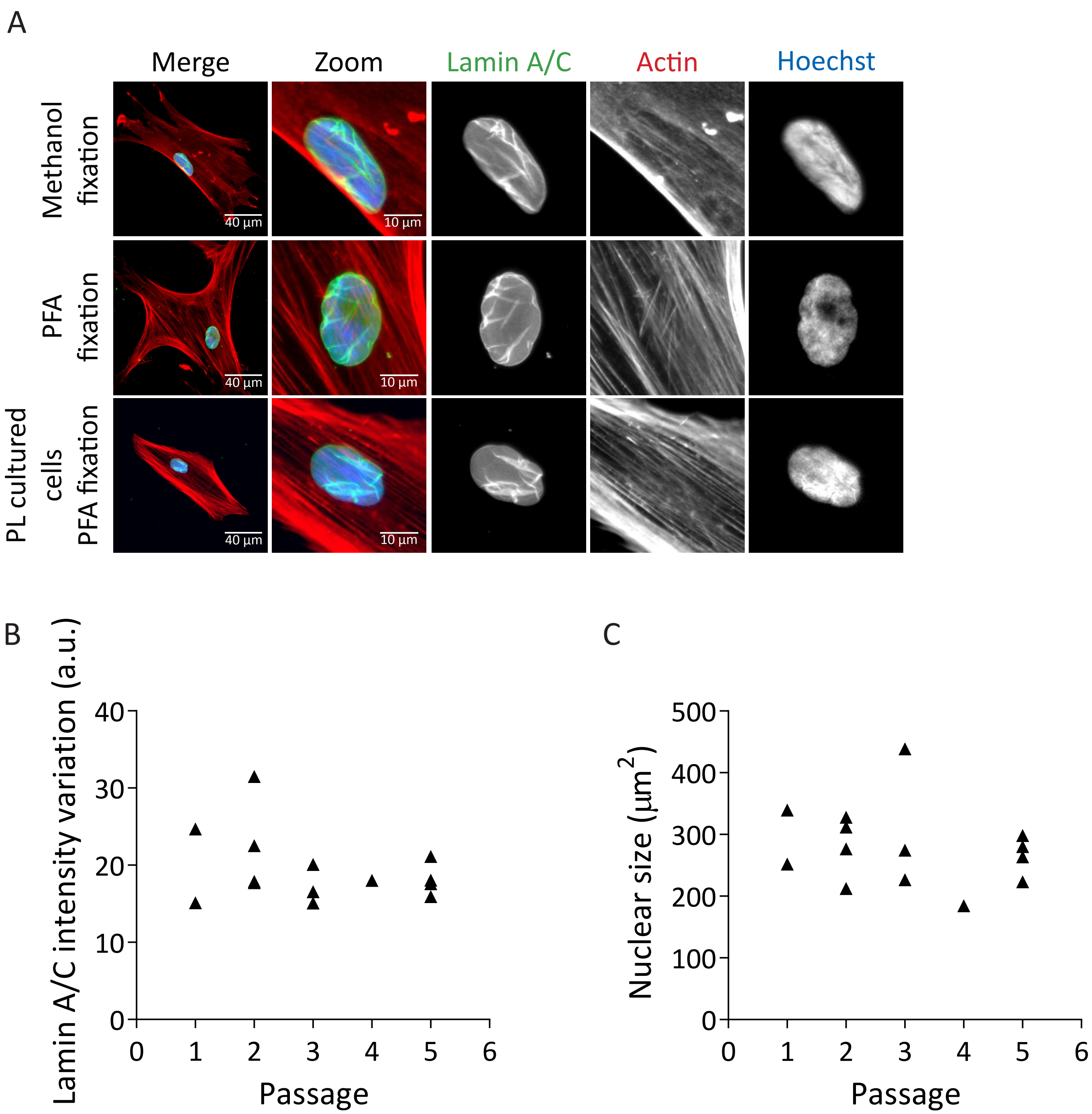
A
Merge
Zoom
Lamin A/C
Actin
Hoechst
Methanol fixation
PFA fixation
PL cultured cells PFA fixation
40 µm
10 µm
B
Lamin A/C intensity variation (a.u.)
Passage
C
Nuclear size (µm²)
Passage

Figure S4

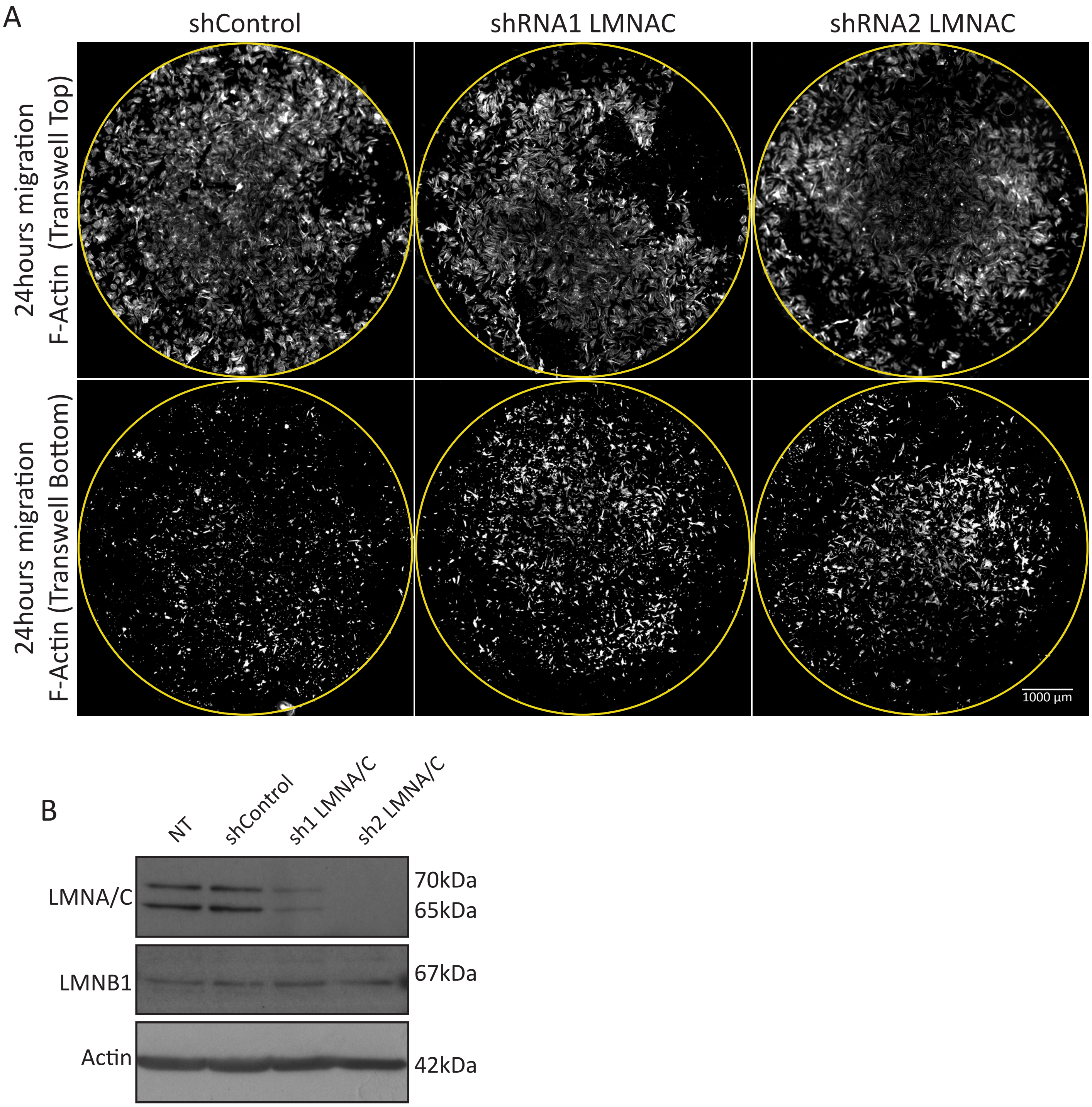
A
shControl
shRNA1 LMNAC
shRNA2 LMNAC
24hours migration
F-Actin (Transwell Top)
24hours migration
F-Actin (Transwell Bottom)
1000 µm
B
NT
shControl
sh1 LMNA/C
sh2 LMNA/C
LMNA/C
70kDa
65kDa
LMNB1
67kDa
Actin
42kDa

Figure S5

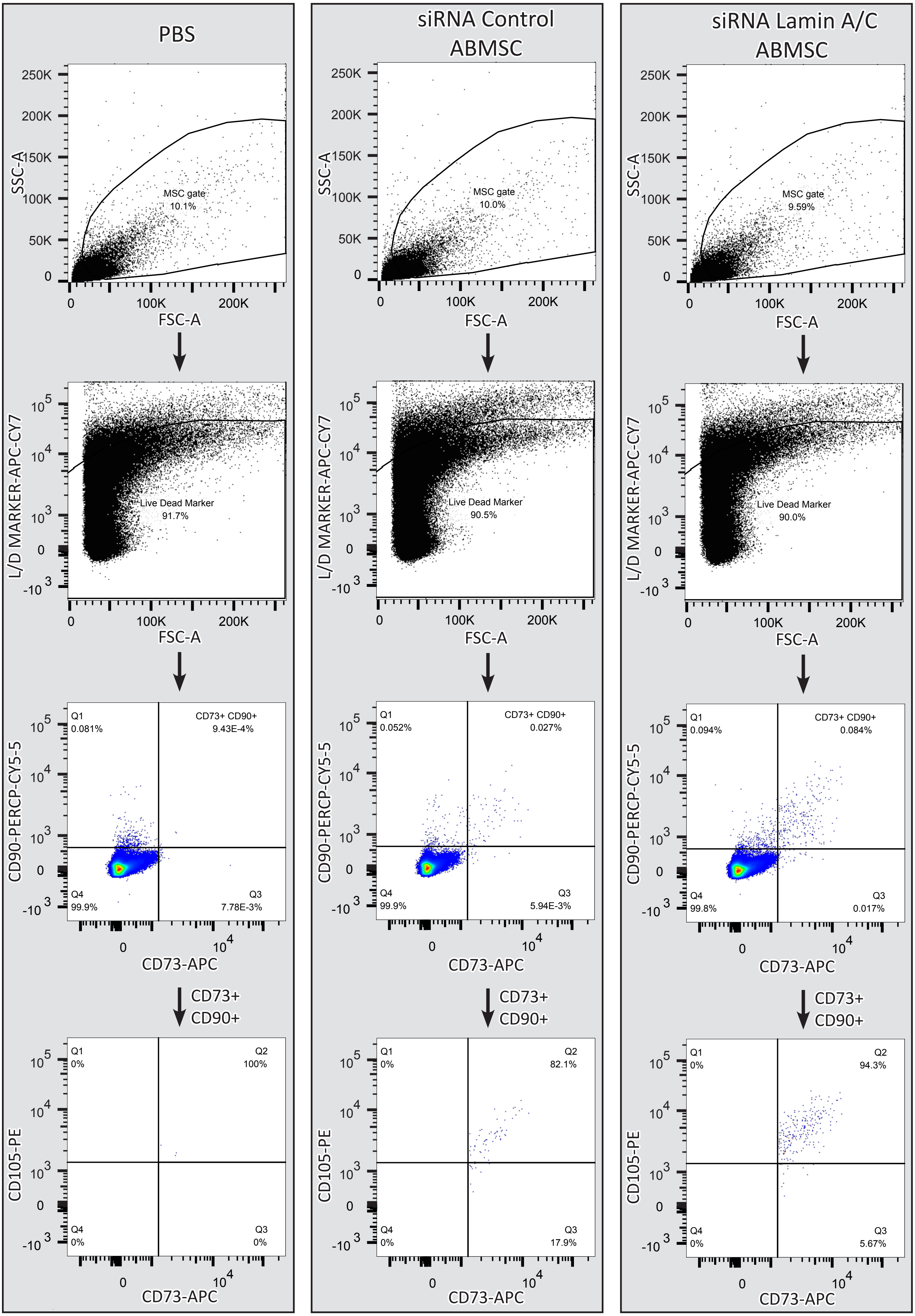
PBS
siRNA Control ABMSC
siRNA Lamin A/C ABMSC
SSC-A
FSC-A
MSC gate
10.1%
10.0%
9.59%
L/D MARKER-APC-CY7
Live Dead Marker
91.7%
90.5%
90.0%
CD90-PERCP-CY5-5
CD73-APC
Q1
0.081%
CD73+ CD90+
9.43E-4%
Q4
99.9%
Q3
7.78E-3%
Q1
0.052%
CD73+ CD90+
0.027%
Q4
99.9%
Q3
5.94E-3%
Q1
0.094%
CD73+ CD90+
0.084%
Q4
99.8%
Q3
0.017%
CD73+
CD90+
CD105-PE
Q1
0%
Q2
100%
Q4
0%
Q3
0%
Q1
0%
Q2
82.1%
Q4
0%
Q3
17.9%
Q1
0%
Q2
94.3%
Q4
0%
Q3
5.67%

Figure S6

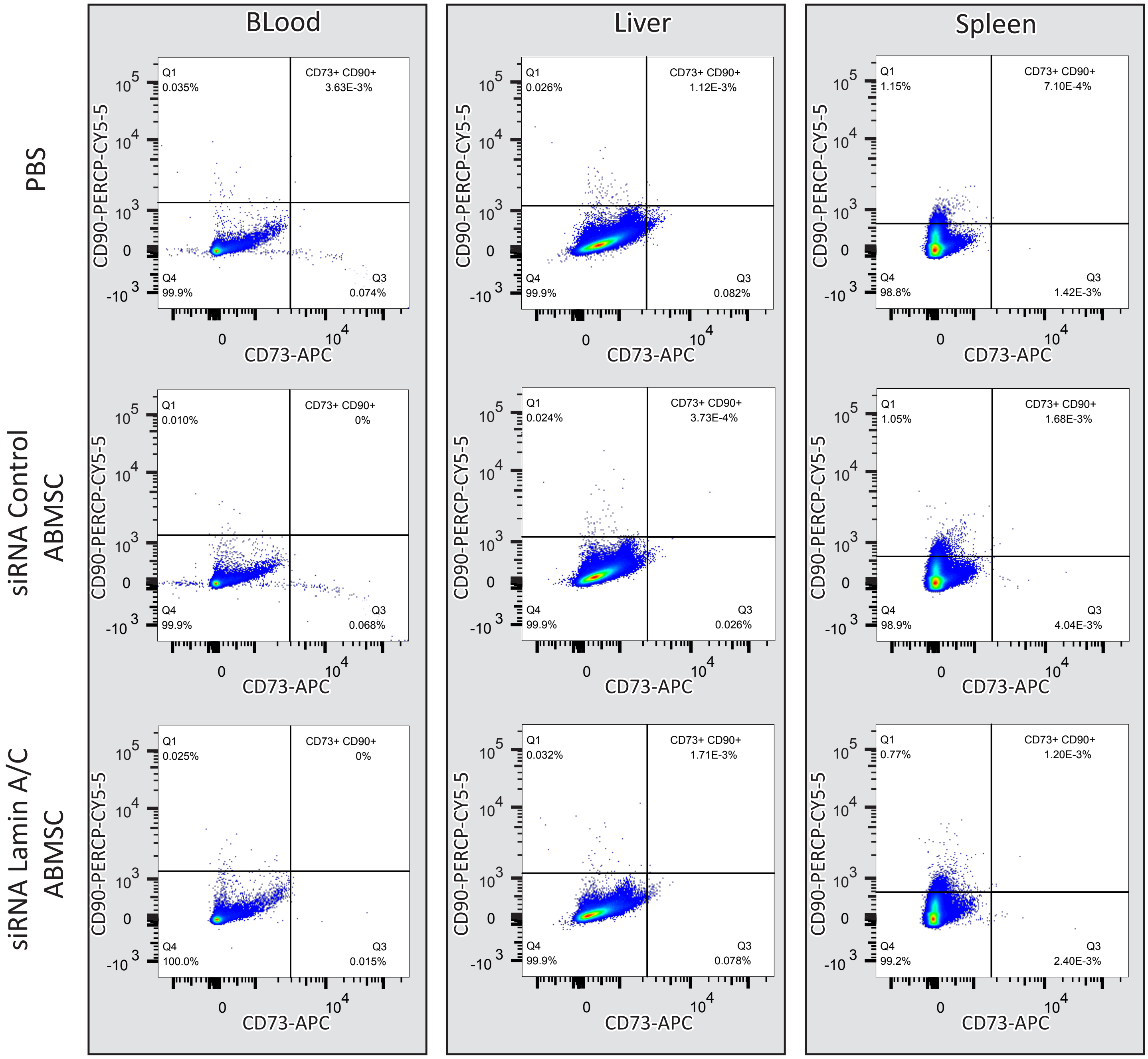

BLood
Liver
Spleen
PBS
siRNA Control ABMSC
siRNA Lamin A/C ABMSC
CD90-PERCP-CY5-5
CD73-APC
Q1 0.035%
CD73+ CD90+ 3.63E-3%
Q4 99.9%
Q3 0.074%
Q1 0.026%
CD73+ CD90+ 1.12E-3%
Q4 99.9%
Q3 0.082%
Q1 1.15%
CD73+ CD90+ 7.10E-4%
Q4 98.8%
Q3 1.42E-3%
Q1 0.010%
CD73+ CD90+ 0%
Q4 99.9%
Q3 0.068%
Q1 0.024%
CD73+ CD90+ 3.73E-4%
Q4 99.9%
Q3 0.026%
Q1 1.05%
CD73+ CD90+ 1.68E-3%
Q4 98.9%
Q3 4.04E-3%
Q1 0.025%
CD73+ CD90+ 0%
Q4 100.0%
Q3 0.015%
Q1 0.032%
CD73+ CD90+ 1.71E-3%
Q4 99.9%
Q3 0.078%
Q1 0.77%
CD73+ CD90+ 1.20E-3%
Q4 99.2%
Q3 2.40E-3%

Figure S7

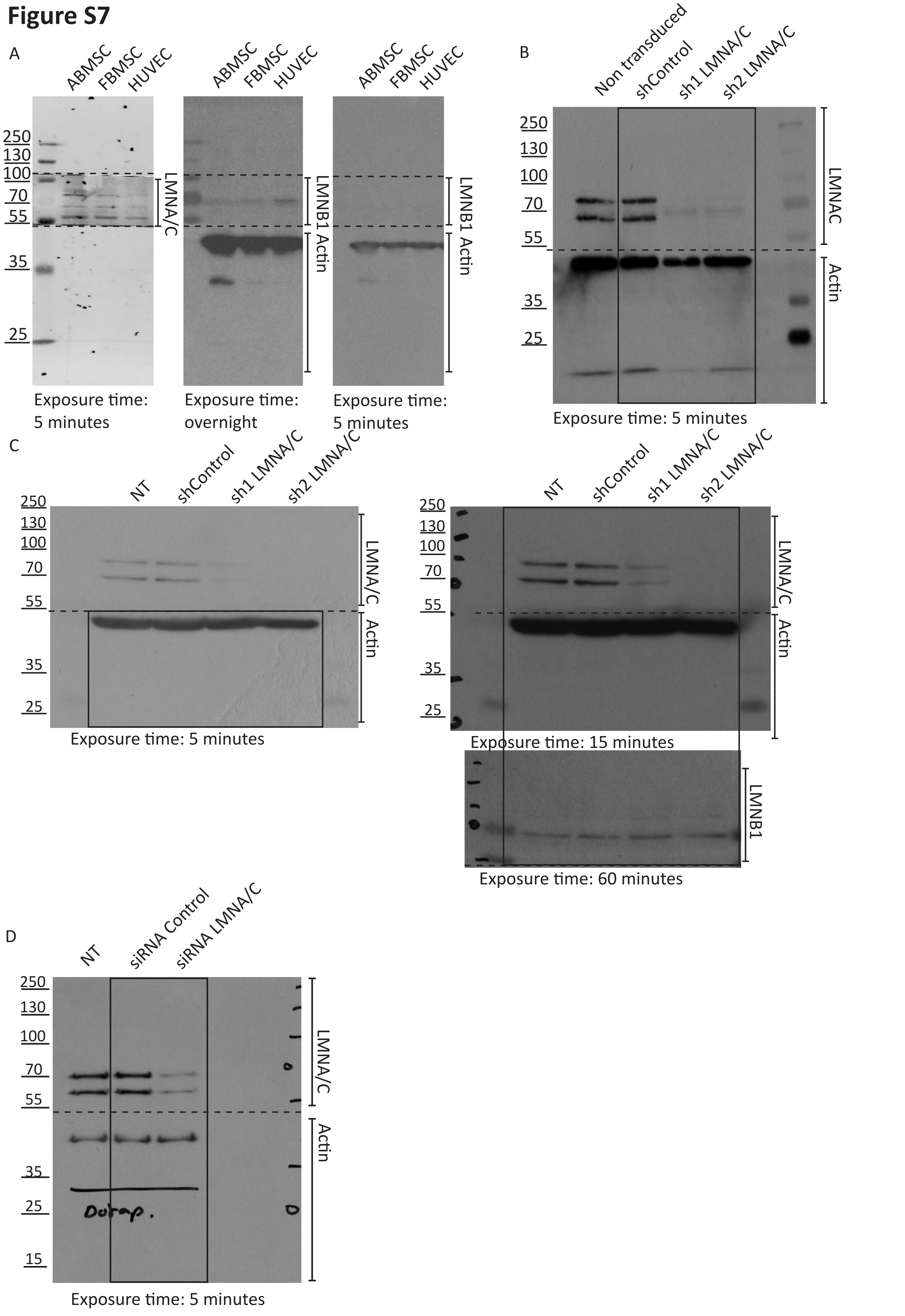
A
ABMSC
FBMSC
HUVEC
250
130
100
70
55
35
25
LMNA/C
LMNB1
Actin
Exposure time: 5 minutes
Exposure time: overnight
Exposure time: 5 minutes
B
Non transduced
shControl
sh1 LMNA/C
sh2 LMNA/C
LMNAC
Actin
Exposure time: 5 minutes
C
NT
shControl
sh1 LMNA/C
sh2 LMNA/C
LMNA/C
Actin
Exposure time: 5 minutes
Exposure time: 15 minutes
LMNB1
Exposure time: 60 minutes
D
NT
siRNA Control
siRNA LMNA/C
250
130
100
70
55
35
25
15
LMNA/C
Actin
Exposure time: 5 minutes

Supplemental Figure and Video legends

Supplemental Figure S1. (a) Individual trajectories of ABMSC, FBMSC, HUVEC and fibroblasts live imaged for 12 hours using brightfield imaging and tracked in ImageJ. n=3 independent experiments, shown are 10 cells per condition. **(b)** Average migration velocity of ABMSCs obtained from donors with varying ages, cultured on 20µg/ml hFN or bFN. 1 donor per condition, analysis includes 15 cells per condition. Spearman correlation test non-significant.

Supplemental Figure S2. (a) Representative widefield IF tilescan images from top and bottom of transwell filters. ABMSC, FBMSC and HUVEC migrated for 24 hours and were subsequently fixed and stained for F-actin. **(b)** Quantification of fully transmigrated ABMSC and FBMSC. Cells were incubated for 48 hours and 7 days and were subsequently fixed and stained for Lamin A/C and F-actin. Widefield images from the bottom of the filter were analysed manually and the number of fully transmigrated cells (stage 3) was calculated as percentage of total protruding cells (stage 2+3). Mean ± s.e.m. n≥3 independent experiments including 30 cells per condition comprising cells derived from at least 3 different donors. Kruskal-Wallis-test followed by an uncorrected Dunn's test, *=p<0.05 **(c)** Quantification of total F-actin at the bottom of the Transwell for ABMSC and FBMSC. Cells were allowed to transmigrate for 48 hours and 7 days and were subsequently fixed and stained for F-actin. F-actin surface area coverage at top and bottom of the Transwell filters was determined from tilescan widefield images covering the entire transwell filter area Cell-body migration (F-actin at Transwell bottom (stage 2+3)) was calculated as a percentage of total F-actin (all stages combined). Mean ± s.e.m. n≥3independent experiments comprising cells from at least 3 different donors. Kruskal-Wallis-test followed by an uncorrected Dunn's test.

Supplemental Figure S3.
(a) Representative widefield immunofluorescence (IF) images of ABMSC stained for Lamin A/C (green), F-actin (red) and Hoechst (blue). Magnified images from regions of interest (Zoom) show detailed structures of the nuclear envelope containing Lamin A/C. Cells were fixed with either methanol (top row) or paraformaldehyde (PFA) (middle and bottom row). Cells were cultured in DMEM with 10% FCS (Top and middle row) or DMEM with 5% platelet lysate (bottom row). Scalebars: 20 µm and 10 µm. **(b,c)** Quantification of the intensity variation **(b)** in a.u. and nuclear size **(c)** based on Lamin A/C IF widefield imaging. Experiments are displayed in order of cell passage number, each data point comprises the analysis of 30 cells, 11 ABMSC donors are included. Spearman correlation test non-significant.

Supplemental Figure S4. (a) Representative widefield IF tilescan images from top and bottom of transwell filters. ABMSC transduced with shRNA Control or shRNA1,2 Lamin A/C migrated for 24 hours and were subsequently fixed and stained for F-actin. **(b)** Western blot analysis of Lamin

A/C, Lamin B1 and actin (loading control) in lysates of ABMSC transduced with shRNA Control or shRNA1,2 targeting Lamin A/C. NT indicates non transduced cells. Images are cropped scans of blots, corresponding whole Western blot scans are shown in Supplemental Figure S7C.

Supplemental Figure S5.
Representative graphs from FACS analysis of siRNA control and Lamin A/C treated ABMSC in murine lung tissue, 14 hours after intravenous administration. From top to bottom the applied gating strategy to quantify the number of ABMSC is displayed. Antibodies were validated to be specific for ABMSC only and did not bind murine cells. Tissues of PBS-injected mice served as a negative control. ABMSC were defined as CD73+, CD90+ and CD105+.

Supplemental Figure S6.
Representative graphs from FACS analysis of siRNA control and Lamin A/C treated ABMSC in murine blood (left), liver (middle) and spleen (right), 14 hours after intravenous administration. Applied gating strategies were the same as for the FACS analysis in lung tissue. Markers against CD73 and CD90 are shown.

Supplemental Figure S7.
Whole scans of Western blots corresponding to data in Figure 4B, 4D, 5A and Supplemental Figure S4. **(a)** Western blot showing Lamin A/C, Lamin B1 and actin (loading control) in lysates of ABMSC, FBMSC and HUVEC. **(b)** Western blot showing Lamin A/C and actin (loading control) in lysates of ABMSC transduced with shRNA Control or shRNA1,2 targeting Lamin A/C. **(c)** Western blot showing Lamin A/C and actin (loading control) in lysates of ABMSC transduced with GFP-control or GFP-Lamin B1. **(d)** Western blot showing Lamin A/C and actin (loading control) in lysates of ABMSC transfected with control or siRNA's against Lamin A/C. NT indicates non transduced cells. Black rectangles indicate the areas used in the figures, dotted lines indicate sliced membranes.

Supplemental movie M1. Migration of ABMSC, FBMSC, HUVEC and fibroblasts. Live widefield microscopy (Zeiss Observer Z1) imaging using EC Plan-Neofluar 10x/0.30 Ph 1 air objective. Frames were taken every 30 minutes for 12 hours at 37°C, 5% CO_2. ABMSC: passage 4, FBMSC: passage 6, HUVEC: passage 5, Fibroblasts: passage 5.

Supplemental movie M2. Z-stack of ABMSC migrating in Transwell assay. ABMSC in 8 µm pore size transwells migrated for 24 hours and were subsequently fixed and stained for F-actin and Lamin A/C. IF Z-stacks of ABMSC were acquired using confocal microscopy (Leica) using a 40x/1.30 oil objective. Cell passage: 4 Total image z-stack comprises 40 µm.
